# Single-molecule multiplexed profiling of protein–DNA complexes using magnetic tweezers

**DOI:** 10.1016/j.jbc.2021.100327

**Published:** 2021-01-23

**Authors:** Lin Liang, Zeyu Wang, Lihua Qu, Wei Huang, Shuang Guo, Xiangchen Guan, Wei Zhang, Fuping Sun, Hongrui Yuan, Huiru Zou, Haitao Liu, Zhongbo Yu

**Affiliations:** 1 State Key Laboratory of Medicinal Chemical Biology, College of Pharmacy, Nankai University, Tianjin, China; 2 Tianjin Key Laboratory of Optoelectronic Sensor and Sensing Network Technology, Institute of Modern Optics, College of Electronic Information and Optical Engineering, Nankai University, Tianjin, China; 3 Central Laboratory of Tianjin Stomatological Hospital, The Affiliated Stomatological Hospital of Nankai University, Tianjin, China

**Keywords:** magnetic tweezers, single-molecule biophysics, multiplexing, protein–DNA interaction, molecular dynamics, DNA methylation, DNA demethylation, BP, base-pair, CGI, CpG island (CGI), dsDNA, double-stranded DNA, SPR, surface plasmon resonance, ssDNA, single-stranded DNA, TET1, ten-eleven translocation enzyme, WLC, worm-like chain model

## Abstract

Epigenetics, such as the dynamic interplay between DNA methylation and demethylation, play diverse roles in critical cellular events. Enzymatic activity at CpG sites, where cytosines are methylated or demethylated, is known to be influenced by the density of CpGs, methylation states, and the flanking sequences of a CpG site. However, how the relevant enzymes are recruited to and recognize their target DNA is less clear. Moreover, although DNA-binding epigenetic enzymes are ideal targets for therapeutic intervention, these targets have been rarely exploited. Single-molecule techniques offer excellent capabilities to probe site-specific protein–DNA interactions and unravel the dynamics. Here, we develop a single-molecule approach that allows multiplexed profiling of protein–DNA complexes using magnetic tweezers. When a DNA hairpin with multiple binding sites is unzipping, strand separation pauses at the positions bound by a protein. We can thus measure site-specific binding probabilities and dissociation time directly. Taking the TET1 CXXC domain as an example, we show that TET1 CXXC binds multiple CpG motifs with various flanking nucleotides or different methylation patterns in an AT-rich DNA. We are able to establish for the first time, at nanometer resolution, that TET1 CXXC prefers G/C flanked CpG motif over C/G, A/T, or T/A flanked ones. CpG methylation strengthens TET1 CXXC recruitment but has little effect on dissociation time. Finally, we demonstrate that TET1 CXXC can distinguish five CpG clusters in a CpG island with crowded binding motifs. We anticipate that the feasibility of single-molecule multiplexed profiling assays will contribute to the understanding of protein–DNA interactions.

DNA dynamics between methylation and demethylation play diverse roles in critical cellular events, such as chromatin coordination and epigenetic regulation of gene expression (1, 2). DNA methylation and demethylation depend on the number and density of CpGs in a CpG island (CGI), methylation states, as well as the flanking sequences of a CpG site (3, 4). CGI recruits proteins of epigenetic enzymes to modulate chromatin structures, determining the bivalency of active or inactive states for either promoting or suppressing gene expressions (5, 6). Thus, DNA-binding epigenetic enzymes are ideal targets for therapeutic intervention (7, 8, 9). However, these targets have been rarely exploited.

There are several conventional methods for studying protein–DNA interactions. NMR, X-ray crystallography, and cryo-electron microscopy have been extensively used for understanding the protein–DNA interactions. Electrophoretic mobility shift assay can estimate at best a K_d_ value to examine the binding affinity between a protein and a simple sequence. Isothermal titration calorimetry provides thermodynamic information about the protein–DNA interactions but consumes large quantities of protein and DNA. At high concentrations of testing samples, the evaluation of binding affinities is sophisticated. Surface plasmon resonance (SPR) and fluorescence methods (10, 11) can examine the kinetics of the interaction, but the binding sites in a DNA sequence are usually challenging to be multiplexed. Chromatin immunoprecipitation offers a high-throughput evaluation of protein–DNA interactions *in vivo*. However, it is not easy to reach the base-pair (bp) resolution for site-specific interactions. Sequencing methods have been recently developed to discover protein–DNA interactions with bp resolution but do not provide information on dynamics (12, 13).

Single-molecule techniques have been used to probe site-specific protein–DNA interactions by mechanically driving DNA-stand separation (14, 15). For example, DNA unzipping experiments using magnetic tweezers demonstrate that interactions between a Tus protein and a DNA sequence of Ter establish a sustained lock at a replication fork with bp resolution (16). A single-molecule counting approach based on a hairpin unzipping assay with magnetic tweezers was recently used to measure the dissociation dynamics of H-NS protein from a single DNA-binding site (17). A mechanical profiling strategy based on magnetic tweezers and optical tweezers was recently developed to precisely and accurately examine the interactions between a DNA with multiplexed recognition sites and its ligands (18). These findings suggest that single-molecule magnetic tweezers are an excellent platform for studying interactions between proteins of epigenetic enzymes and multiplexed DNA binding sites.

Here, we developed a single-molecule approach that allows multiplexed profiling of protein–DNA complexes using magnetic tweezers. To detect protein–DNA interactions, we designed a DNA hairpin containing multiple binding sites of a target protein. When a DNA hairpin is unzipping, strand separation pauses at the positions bound by a protein. We can thus measure site-specific binding probabilities and dissociation time directly. Because the TET1 CXXC domain binds to CpG with C being nonmethylated, methylated, or hydroxymethylated (19), we take the CXXC domain of TET1 protein as an example showing how TET1 CXXC binds multiple CpG motifs with various flanking nucleotides or different methylation patterns in an AT-rich DNA sequence. We can determine probabilities and dissociation time of CXXC-interacting events on DNA at nanometer resolution. With the DNA containing multiplexed binding sites, we show that TET1 CXXC prefers G/C flanked CpG motif over C/G, A/T, or T/A flanked ones, which is previously unknown. CpG methylation strengthens TET1 CXXC recruitment but has little effect on dissociation time. In a proof of concept, we finally demonstrate that TET1 CXXC can distinguish five CpG clusters in a CGI with crowded binding motifs. *Via* multivalent interactions, CpG clusters extend the dissociation time of TET1 CXXC 10x longer than that at a single CpG site. Our results suggest the feasibility of single-molecule multiplexed profiling assays using magnetic tweezers for investigating the interactions between a protein and DNA with diverse binding motifs.

## Results

### Single-molecule profiling of specific protein–DNA complexes

**Figure 1:**
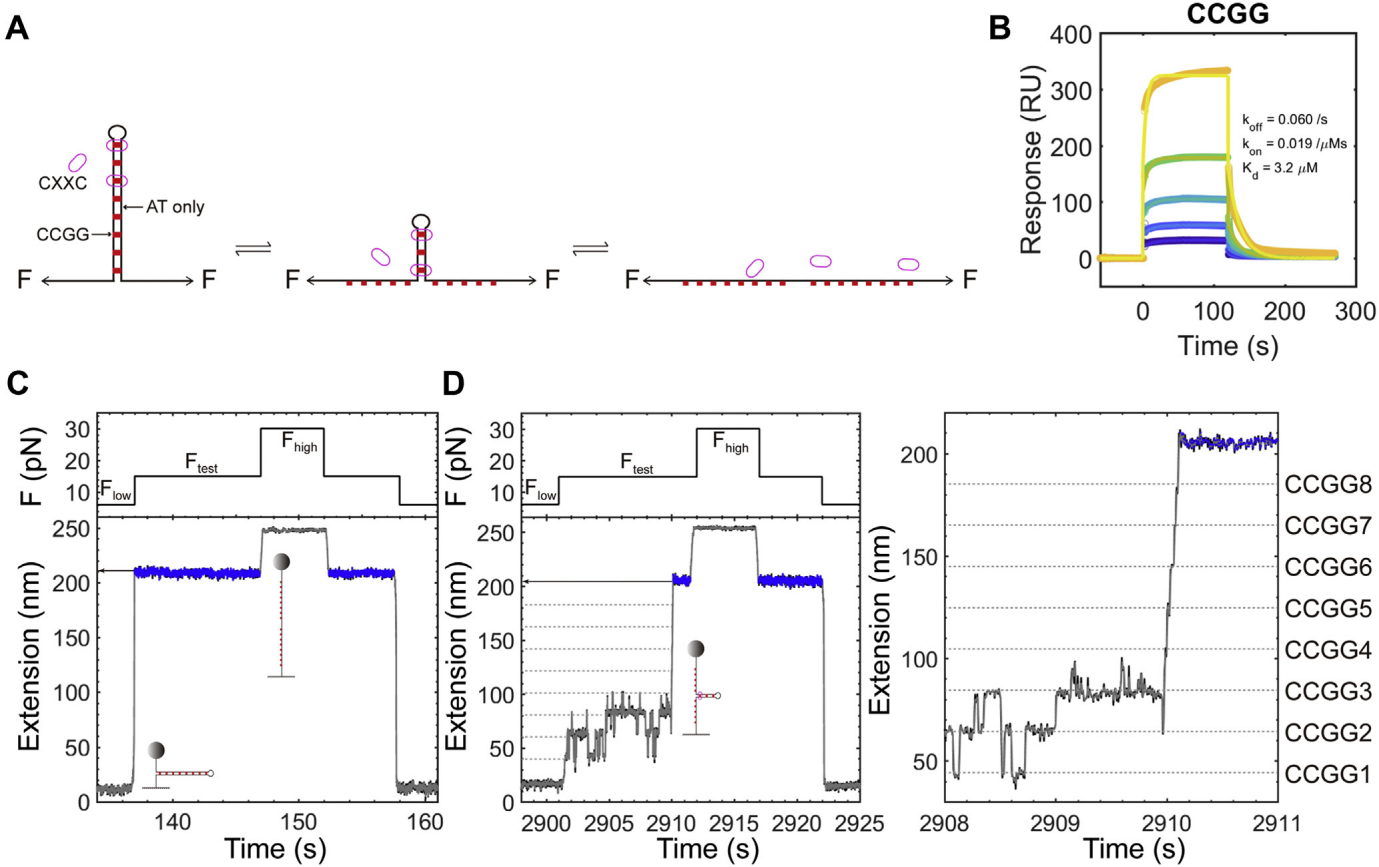
**Setup and detection of interactions between a protein and its specific DNA-binding sites.***A*, Schematic for single-molecule profiling of a CXXC protein on a CCGG DNA. *Red blocks* indicate CCGG sites in the hairpin stem with ovals representing a CXXC protein. *B*, SPR for TET1 CXXC binding a CCGG dsDNA. K_d_ = 3.2 μM. TET1 CXXC concentrations are 0.5 μM, 1 μM, 2 μM, 4 μM, and 8 μM. The biotin-modified dsDNA contains a CCGG site. The buffer for SPR measurement contains 10 mM Tris (pH 7.4), 100 mM NaCl, 50 μM ZnCl_2_, 1 mM DTT, 3% glycerol, and 0.005% Tween 20. *C*, The force manipulation and the corresponding changes in extension of the CCGG hairpin without protein. The protocol has three forces, F_low_ = 6 pN, F_test_ = 14.75 pN, and F_high_ = 30 pN. *Blue color* highlights the extension where the CCGG hairpin is fully open. *D*, Detection of interactions between TET1 CXXC and CCGG sites. *Dotted lines* indicate the positions of eight evenly distributed CCGGs in the hairpin stem (Zoomed in for details in the *right panel*). [TET1 CXXC] = 3 μM. Sampling rate = 200 Hz.

We designed a CCGG hairpin to directly measure the interactions between a CpG-binding protein and its targeting DNA. The 194 bp stem of the hairpin construct has eight evenly distributed CCGG motifs with 18 bp spacers between two neighbors (Figs. 1*A* and S1). To specifically probe CpG-binding events on CCGG sites, we generate the interspersed spacers of 18 bp with A and T only using a random number function in MatLab and minimize the formation of secondary structures by mFold (See DNA sequences in Table S1) (20, 21). We commercially synthesized the TET1 CXXC of 46 amino acids (Table S2) and validated its activities using SPR (Fig. 1*B* and S2). The K_d_ for TET1 CXXC binding a CCGG DNA is 3.2 μM by SPR (Fig. 1*B*), similar to the K_d_ of 3.1 ± 0.4 μM in the literature (22). Using single-molecule magnetic tweezers, we unzip the hairpin stem above melting forces. CXXC-binding events on CCGG sites can block the strand separation of the double-stranded DNA (dsDNA) from the upstream to the downstream. By measuring signals of interruptions with precise positions and durations upon breaking the complex of protein–DNA, we can site-specifically interpret the dynamics of CXXC dissociation from a CCGG motif in real time.

We employed a force-manipulating protocol of three levels in single-molecule profiling assays (Experimental procedures). At a low force (F_low_), *e.g.*, 6 pN, the stem of the hairpin construct remains a dsDNA conformation (Fig. 1*C*, *left, bottom* cartoon). At a testing force (F_test_) above the critical melting force of the hairpin stem, *e.g.*, 14.75 pN, the dsDNA of the stem cooperatively unzips to be single-stranded DNA (ssDNA) without pauses (Fig. 1*C*, *left, top* cartoon). For the CCGG hairpin construct, the critical force of unzipping the stem is 11.9 pN ± 0.6 pN (Mean ± sd, n = 54, Fig. S1*D*). At a high force (F_high_), *e.g.*, 30 pN, the melted hairpin further extends, assuring the complete dissociation of bound proteins. We next decrease the force from F_high_ to F_test_, then to F_low_, finishing a round of the protocol. At [CXXC] = 3 μM, we probe the binding events of CXXC on the CCGG hairpin stem using a force manipulating protocol with F_low_ = 6 pN, F_test_ = 14.75 pN, and F_high_ = 30 pN (Fig. 1*D*). The pausing signals in a single trace at F_test_ explicitly show eight states that are evenly spaced along with the DNA extension.

**Figure 2:**
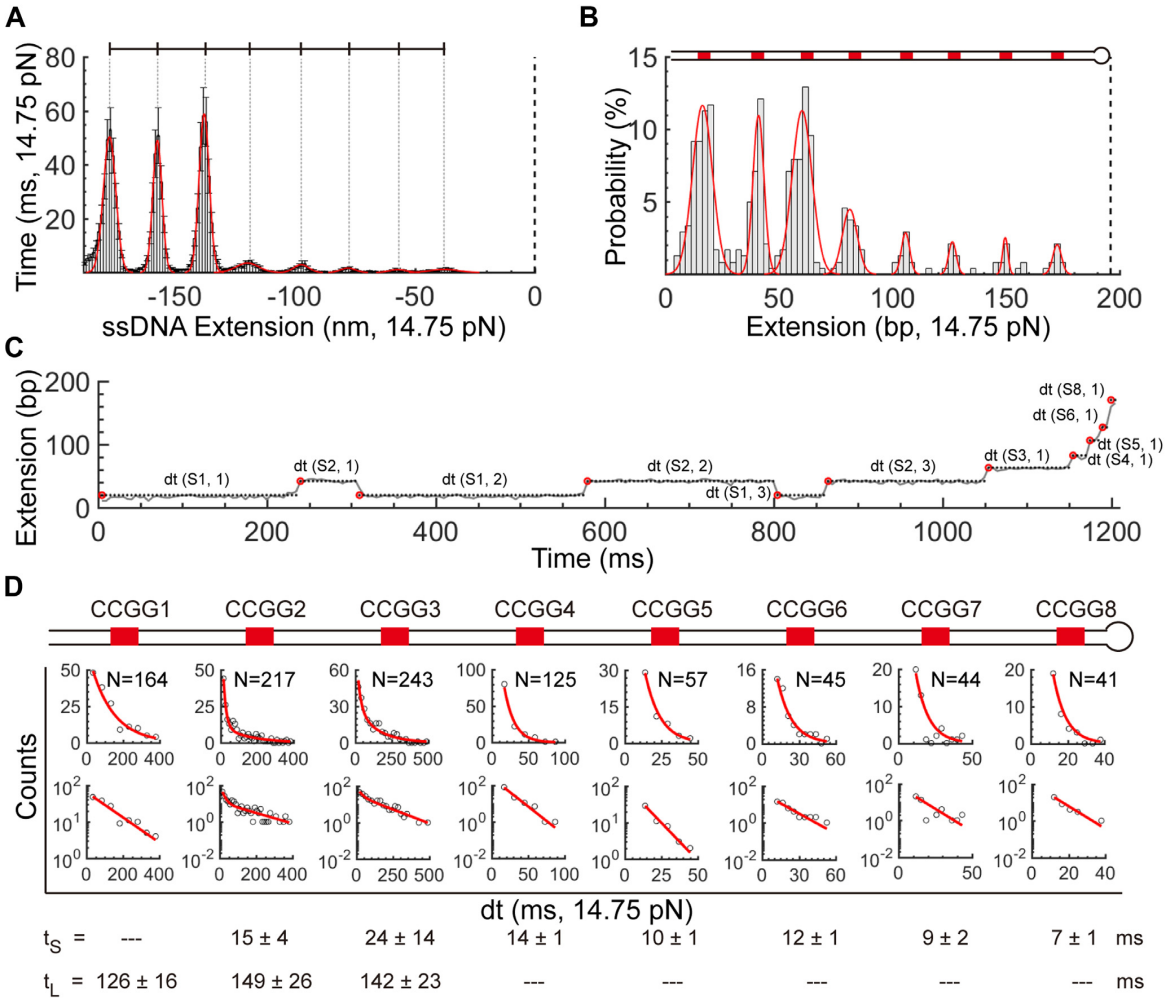
**Time and probability analysis of TET1 CXXC binding the CCGG hairpin.***A*, Histograms of 101 force-jumping traces for TET1 CXXC binding the CCGG hairpin. Bin size = 1.2 nm. Heights are means with standard errors (se). *Thin dotted lines* indicate CCGG positions. The *thick dotted line* marks the zero position where the CCGG hairpin is fully open. *B*, Histograms for binding probabilities of TET1 CXXC along the CCGG hairpin stem. Bin size = 2.46 bp. *Red curves* are Gaussian fittings. The *dotted line* indicates the loop position of the CCGG hairpin. The *top* cartoon illustrates the hairpin. N = 240 traces. *C*, Dwell time measurements. *Red circles* mark the starting point of dwell time. *Dotted lines* represent the time duration. In the notation of dt(Sn, m), n stands for a CCGG site and m for the sequential index of dwell time. *D*, Histograms of dwell times fitted by exponential functions. Distributions are shown in both normal and log scales. *Red curves* are either single- or double-exponential fittings. Fitting results are shown with se where t_S_ stands for the coefficients of short-lived events and t_L_ for long-lived events (Detailed fitting results in Table S3).

The evenly distributed footprints of TET1 CXXC on the CCGG hairpin can serve as a ruler to convert the DNA extension from nanometer to bp. Using the fully opened extension of the CCGG hairpin as the reference position of zero at F_test_, we take 101 traces to build histograms along with the ssDNA extension (Fig. 2*A*). We divide the peak heights by the sampling rate (200 Hz in this case) to obtain the overall dwell time on a specific site. Averaging the 101 histograms with the same bins (Here bin size = 1.2 nm), we observe eight peaks that exactly matched with the eight CCGG sites in the hairpin stem as designed. We theoretically calculate the extension differences between adjacent CCGG sites using a worm-like chain model of Marko–Siggia (Experimental procedures, Equation 1) at a salt condition of 100 mM NaCl (23, 24). By comparing the theoretically calculated results with the peak-to-peak distances measured in the experiments of the CCGG DNA, we find that the hairpin fork preferentially pauses at the expected high-affinity CXXC-binding motifs of CCGG within a relative error of 4% at F_test_ = 14.75 pN. We thus convert the axis of DNA extension from nanometer to bp using Equation 1.

Binding probabilities of TET1 CXXC at the eight CCGG sites show two distinct modes, either high or low. To locate a protein-binding event at a CpG site, we check whether there is a pause (>10 ms) within ±11 bp around the CpG site at F_test_. We count only once for multiple visits at the same CCGG site. After dividing the number of pauses at a CCGG site by the total rounds of force manipulations, we obtain the site-specific binding probabilities (Fig. 2*B*, bin size = 2.46 bp, n = 240 traces). The binding events at the three upstream CCGGs show high probabilities of >10%, while the binding probabilities at the other five downstream CCGGs are as low as < 5% (Fig. 2*B*). Since the eight CCGGs are the same, which TET1 CXXC cannot distinguish, the two distinct modes may reflect the asymmetry of the fork-like DNA hairpin construct. During target search, a protein can bind DNA nonspecifically and laterally diffuse on DNA until it dissociates or lands on a target site (15, 25). The upstream CCGG sites are close to the fork handles of dsDNA, which may thus influence the TET1 CXXC-binding probability by nonspecific attraction.

Analysis of pausing time reveals the site-specific dynamics of TET1 CXXC dissociation forced by DNA-strand separation. Considering the sampling rate of 200 Hz and its Nyquist frequency, we only measure pauses of >10 ms (Fig. 2*C*). We site-specifically build histograms of the pausing time for each CCGG, *i.e.*, covering ±11 bp around a CpG site (See Experimental procedures for detailed data analysis). At the five downstream CCGG sites (CCGG4-8), we find that a single-exponential function fits the histograms well (Fitting equations in Experimental procedures), revealing an expected value of pausing time to be ∼14 ms at F_test_ = 14.75 pN (Fig. 2*D*, bottom, and Table S3 for fitting goodness). In contrast, a double-exponential function fits the two histograms well at upstream CCGG2 and CCGG3, in which the short pausing time (t_S_) is at the same level as that at CCGG4-8, ∼14 ms. However, the long pausing time (t_L_) of ∼140 ms is 10x more prolonged than the short one (Fig. 2*D*, bottom, F_test_ = 14.75 pN). At CCGG1, the first TET1 CXXC-binding site to be unzipped in our single-molecule mechanical manipulation, we only find the long pausing time of 126 ms, almost identical to t_L_ = 140 ms at CCGG2-3 within error (Fig. 2*D*, bottom, F_test_ = 14.75 pN). Because the downstream CCGG4-8 only shows the short pausing time of t_S_ = ∼ 14 ms upon unzipping the dsDNA at the presence of TET1 CXXC (3 μM), we attribute this short-lived process to the events of disrupting a simple CXXC-CCGG complex by a testing force (14.75 pN). When unzipping the upstream dsDNA, a long pausing time of t_L_ = ∼ 140 ms indicates a different event from that merely breaking a CXXC-CCGG complex. The asymmetry of the fork-like hairpin construct may explain the event with a long pausing time. TET1 CXXC may simultaneously interact with the CCGG sites in the upstream hairpin stem, and the nearby fork handles of dsDNA nonspecifically, resulting in a long pausing time for a complex dissociation event.

To test whether protein-mediated nonspecific interactions between the upstream hairpin stem and fork handles cause the bimodal effect, *i.e.*, two different modes of the protein dissociation under forces, we further examined this effect on the CCGG hairpin using a concentration of [TET1 CXXC] = 0.8 μM, which is ∼4x lower than the previous concentration of 3 μM. At CCGG2-3, overall dwell time and binding probability dramatically decrease compared with that at CCGG1 at the lowered concentration of TET1 CXXC and become comparable with that at CCGG4-8 (Fig. S3). The concentration responses support our assumption of a nonspecific binding mechanism, allowing us to adjust the ligand concentration to suppress the bimodal effect.

We also find that the bimodal effect depends on the binding motif of TET1 CXXC. We mutate the CCGG1-3 of the CCGG hairpin to be CATG. As shown in Figure S4, the bimodal effect disappears in the CATG hairpin construct, supporting the hypothesis of nonspecific interactions between hairpin upstream stem and fork handles. In addition, we make a new construct of dense CCGG hairpin by adding three more CCGG sites at the upstream stem of the CCGG hairpin (Fig. S5). Such an arrangement creates two domains with either high or low density of CCGG sites in the hairpin stem. The bimodal effect remains at the upstream hairpin stem with a high density of CCGG sites, while the binding event is almost nondetectable at the downstream hairpin stem with a low density of CCGG sites. This observation could also be explained by the assumption of nonspecific interactions between upstream hairpin stem and fork handles. The positions of binding motifs play an essential role in the bimodal effect. The five CCGG sites in the downstream hairpin stem are beyond the influence of nonspecific interactions, like that in the CCGG hairpin under the same experimental conditions. Overall, the current set of data supports our assumption of nonspecific interactions regarding the bimodal effect.

### Multiplexed profiling of single proteins on different DNA targets

**Figure 3:**
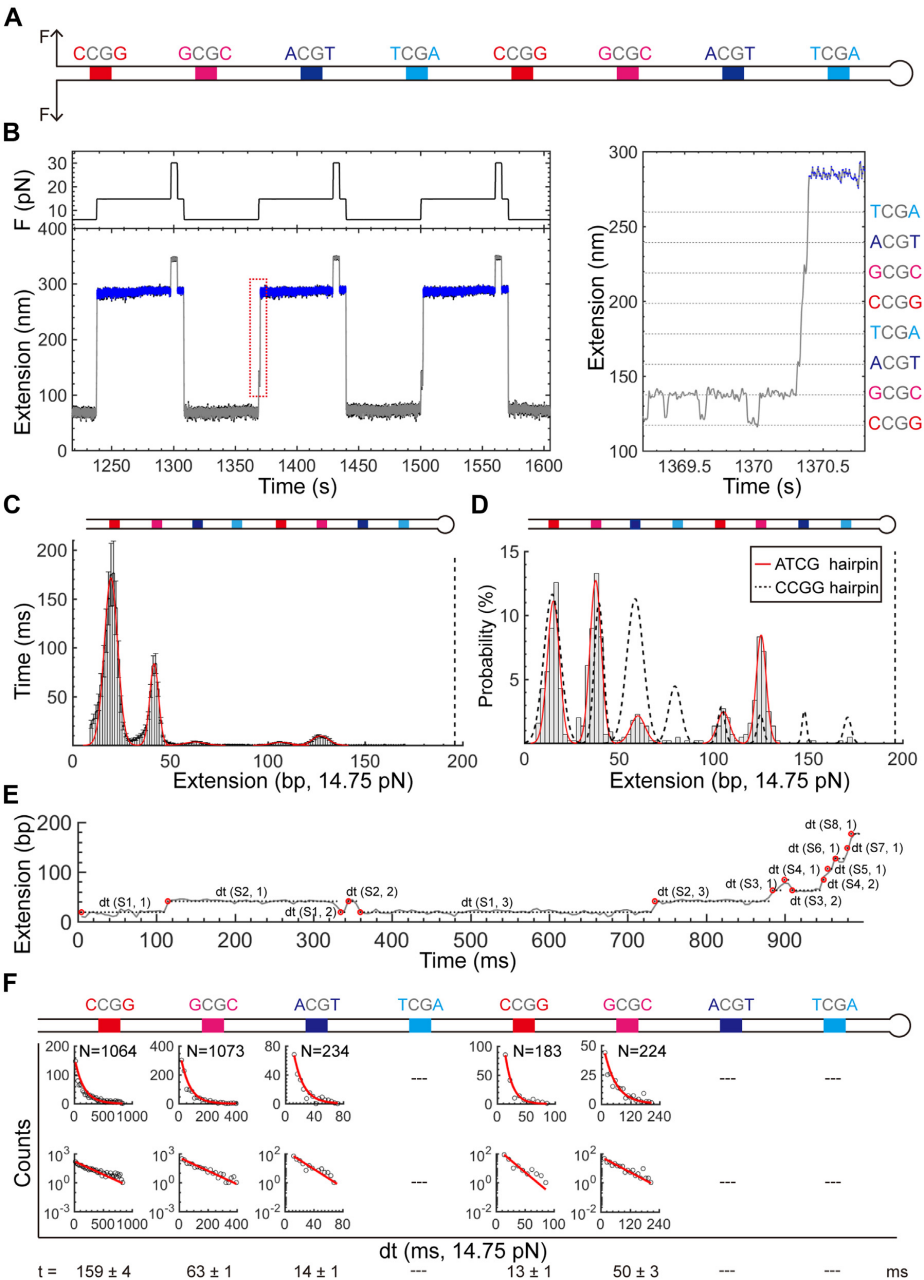
**Multiplexed profiling of TET1 CXXC binding the ATCG hairpin.***A*, Scheme of the ATCG hairpin. Eight CpG sites are flanked by multiplex nucleotides (Colored). *B*, Repetitive force-jumping assays to examine the binding of TET1 CXXC on the ATCG hairpin. The *red dotted window* is zoomed in for details on the *right*. *Dotted lines* mark the expected positions for the CpG sites. *C*, Histograms of 211 force-jumping traces for TET1 CXXC binding the ATCG hairpin. Bin size = 1.2 nm. Heights are means with se. The *dotted line* at zero marks the loop position of the ATCG hairpin. The cartoon at top illustrates the CpG sites with multiplexed flanking nucleotides in the ATCG hairpin. *D*, Histograms for binding probabilities of TET1 CXXC along the ATCG hairpin stem. Bin size = 2.46 bp. *Red curves* are Gaussian fittings. *Dotted curves* indicate binding probabilities from the CCGG hairpin. N = 445 traces. *E*, Dwell time measurements with the same style as that in Figure 2*C*. *F*, Histograms of dwell times fitted by a single-exponential function. Distributions at normal and log scales are shown. The fitting results are listed at the bottom with standard errors (Detailed fitting results in Table S3).

To develop a multiplexed profiling method for probing interactions between a CXXC protein and its different targeting sites, we designed an ATCG hairpin to examine how the immediate neighboring nucleotides of a CpG site affect TET1 CXXC’s binding activity. Although dinucleotides targeted by TET1 CXXC can be either CpG or CpH (H for any base other than G), TET1 CXXC has a preference of CpG over CpH (22). Like the CCGG hairpin construct, the ATCG hairpin has eight CpG sites evenly distributed in the stem of 194 bp (Fig. 3*A*). Unlike the CCGG hairpin with only C/G flanked CpGs, the eight CpGs in the ATCG hairpin have the two 5′ and 3′ neighboring nucleotides to be C/G, G/C, A/T, and T/A, forming subsequent sites of CCGG, GCGC, ACGT, and TCGA, which are duplicated once in the downstream stem (Fig. 3*A* and Table S1). Considering the bimodal effect is position-sensitive along the hairpin stem in the CCGG construct, we have remained the CCGG1 and CCGG4 unchanged when preparing the ATCG hairpin. CCGG1 and CCGG4 thus serve as internal controls when performing a site-specific comparison between CCGG and ATCG hairpins. Spacers among the eight CpGs of the ATCG hairpin are AT only as that in the CCGG hairpin.

Multiplexed profiling assays reveal that TET1 CXXC interrupts the unzipping of the ATCG hairpin by binding at the CpG sites. We performed the single-molecule profiling assays under the same experimental conditions as that for the CCGG hairpin. Without TET1 CXXC, we find no pauses (Fig. S6). With TET1 CXXC, we frequently observe pausing events at F_test_ = 14.75 pN (Fig. 3*B*, *left*). The pausing positions at F_test_ precisely show the footprints of TET1 CXXC on the sites of CpGs as designed above (Fig. 3*B*, *right*).

TET1 CXXC’s pausing events show overall longer durations at C/G and G/C flanked CpGs than that at the A/T and T/A flanked ones in the ATCG hairpin. Along with the extension of the ATCG hairpin stem, we take averages of 211 histograms from individual traces at F_test_ = 14.75 pN. The overall pausing durations at the upstream CpGs of the ATCG hairpin are more prolonged than that at the downstream CpGs (Fig. 3*C*), a similar phenomenon happening in the CCGG hairpin. Unlike the CCGG hairpin, we observe that accumulative durations are close to zero at A/T and T/A flanked CpGs in the ATCG hairpin (Fig. 3*C*). The site-specific pausing durations thus show that TET1 CXXC prefers to bind C/G and G/C flanked CpGs over A/T and T/A flanked ones, similar to that for the CXXC domain of CFP1 (4).

Binding probabilities also validate the preference of TET1 CXXC on CG flanked CpGs over the AT flanked ones. At either the upstream or the downstream stem, binding probabilities at CG flanked CpGs are higher than their neighboring AT flanked ones (Fig. 3*D*, bars and red curves, n = 445 traces). Binding probabilities of TET1 CXXC at the first two upstream CCGG and GCGC sites are higher than the rest sites. The binding probabilities at the upstream ACGT are higher than that at the downstream one (3% vs. 0%). Such a bimodal phenomenon is like that in the CCGG hairpin.

Furthermore, we find that TET1 CXXC prefers the GCGC motif over the CCGG motif. At the downstream stem of the ATCG hairpin, we observe an outstanding binding probability at the GCGC site in contrast to its neighboring CCGG site (9% versus 3%). Because the experimental conditions are the same between the CCGG hairpin and the ATCG hairpin, we compare the binding probabilities along the eight CpGs in the two hairpins (Fig. 3*D*, red solid curves for the ATCG hairpin, black dotted curves for the CCGG hairpin). By comparison at the downstream GCGC site of the ATCG hairpin to its corresponding place in the CCGG hairpin, TET1 CXXC indeed shows a much higher preference to GCGC over CCGG (9% vs. 2%). At the CpG1-2 sites in the ATCG hairpin, TET1 CXXC-binding probabilities are identical to that in the CCGG hairpin, assuring a fair comparison. In addition, we find that TET1 CXXC binds CCGG more often than either ACGT or TCGA at both upstream and downstream stem (Fig. 3*D*). The binding probabilities at the two TCGA sites in the ATCG hairpin are close to zero. Such comparison again supports our finding that TET1 CXXC prefers CCGG over AT flanked CpGs, as shown above.

Analysis of pausing time further supports the G/C preference of TET1 CXXC over C/G, A/T, or T/A flanking sequences. We measure the pausing times for each CpG site in the ATCG hairpin (Fig. 3*E*). All the available histograms can be fitted well with a single-exponential function (Fig. 3*F*). The expected pausing time from fittings at the upstream ACGT and the downstream CCGG sites is ∼14 ms, identical to the short pausing time in the CCGG hairpin, suggesting a simple dissociation event of TET1 CXXC from DNA. The expected pausing time at the upstream CCGG site of the ATCG hairpin is 159 ms, close to a long pausing time of 126 ms in the CCGG hairpin, indicating a complex dissociation event. Interestingly, the pausing times at either the upstream or the downstream GCGC sites are 50 ∼ 60 ms, about 4x longer than the dissociation time of TET1 CXXC from a CCGG site. The pausing time analysis thus shows that TET1 CXXC prefers G/C flanked CpGs over others with a rank of GCGC > CCGG > ACGT ∼ TCGA.

**Figure 4:**
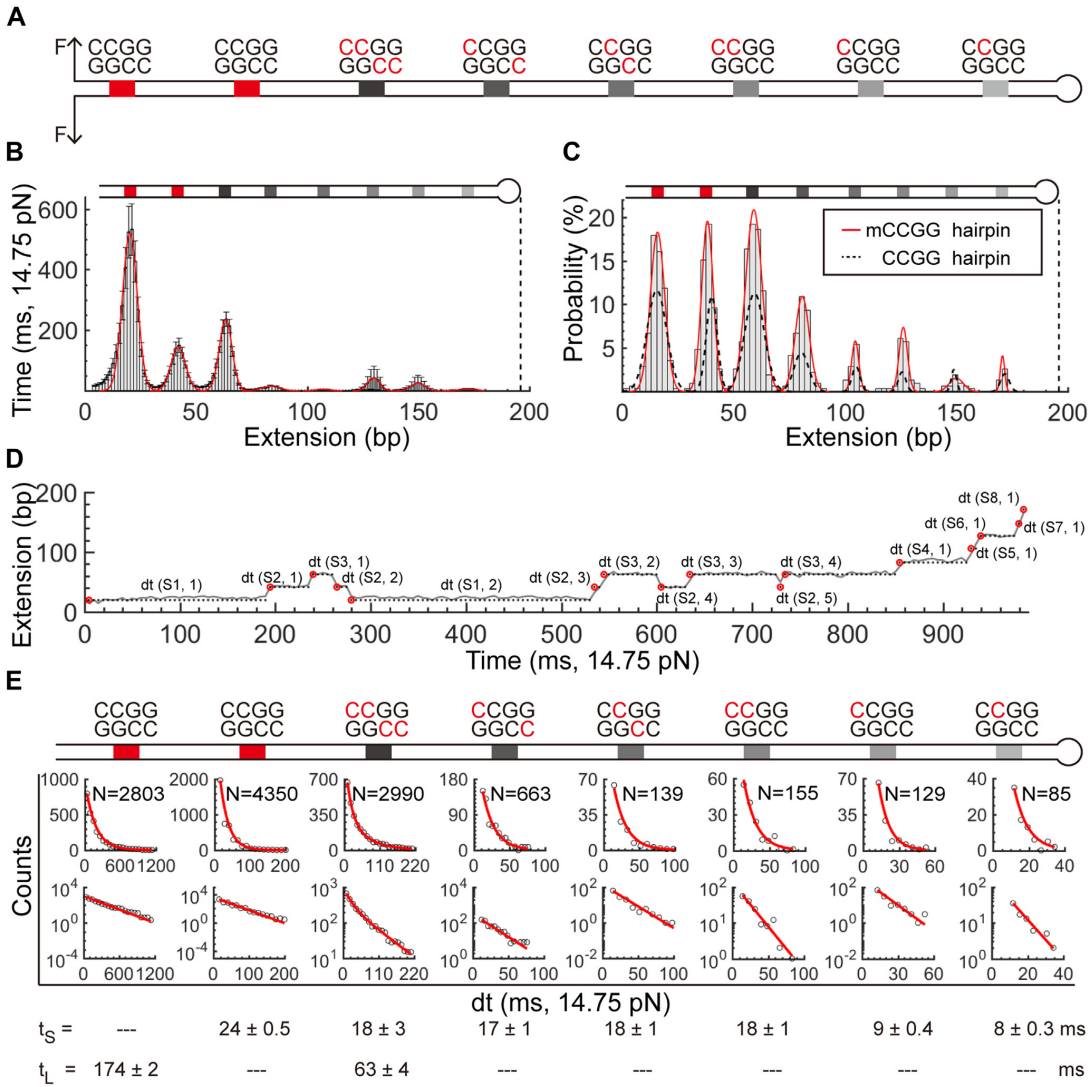
**Multiplexed profiling of TET1 CXXC binding the mCCGG hairpin.***A*, Scheme of the mCCGG hairpin. *Red* C marks the methylation sites. *B*, Histograms of 177 traces for TET1 CXXC binding the mCCGG hairpin. The data treatment and experimental conditions are the same as those for the ATCG hairpin. *C*, TET1 CXXC-binding probabilities on the mCCGG hairpin (Bars and *red* Gaussian curves, N = 312 traces) and the CCGG hairpin (*Dotted curves*). *D*, Dwell time measurements with the same style as that in Figure 2*C*. *E*, Histograms of dwell times. Distributions at normal and log scales are shown. The fitting results are given with standard errors (Detailed fitting results in Table S3).

To further explore TET1 CXXC’s targeting sequences, we methylated the CCGG hairpin at cytosines to examine how TET1 CXXC dynamically interacts with fully methylated or hemimethylated CpGs with various methylation patterns. SPR assays show that TET1 CXXC can bind to a CpG with C being nonmethylated or methylated (Fig. 1*B* and S2). In the eight CCGGs, we leave CCGG1-2 unmethylated and modified CCGG3 to $\begin{array}{l} mCmCGG\\ GGmCmC \end{array}$, CCGG4 to $\begin{array}{l} mCCGG\\ GGCmC \end{array}$, CCGG5 to $\begin{array}{l} CmCGG\\ GGmCC \end{array}$, CCGG6 to $\begin{array}{l} mCmCGG\\ GGCC \end{array}$, CCGG7 to $\begin{array}{l} mCCGG\\ GGCC \end{array}$, and CCGG8 to $\begin{array}{l} CmCGG\\ GGCC \end{array}$ (Experimental procedures, Table S1). The methylated CCGG hairpin (mCCGG hairpin) thus contains six mCCGG sites with different methylation patterns and two CCGG sites as nonmethylation controls (Fig. 4*A*).

Under the same experimental conditions as that for the CCGG hairpin, we run multiplexed profiling assays for TET1 CXXC on the mCCGG hairpin. The TET1 CXXC’s overall pausing times in the mCCGG hairpin show an entirely different pattern from that without TET1 CXXC (Fig. S7) and that in the CCGG hairpin (Fig. 2*A*), suggesting an effect due to the interactions between TET1 CXXC and methylated CCGGs (Fig. 4*B*, n =177). After calculating the binding probabilities of TET1 CXXC on the mCCGG hairpin, we find a bimodal effect, like that on the CCGG hairpin, but with almost doubled binding chances (Fig. 4*C*, bars and red curves for the mCCGG hairpin, black dotted curves for the CCGG hairpin). The binding probability at mCCGG7 ($\begin{array}{l} mCCGG\\ GGCC \end{array}$) is an exception, showing the same likelihood as its related site in the CCGG hairpin. The hemimethylated mCCGG7 has just one methylated cytosine in two strands and beyond the CpG motif, giving a negligible effect on TET1 CXXC’s binding probability. Other than the methylated sites in the mCCGG hairpin, the binding chances at the nonmethylated CCGG1-2 also increase compared with their corresponding sites in the CCGG hairpin, indicating that enhanced recruitment of TET1 CXXC by the methylated CCGG motifs boosts binding events at their neighbors.

TET1 CXXC binds DNA with a motif of three amino acid residues, ^615^SHQ^617^. H^616^ can form two hydrogen bonds with a CG pair. However, S^615^ and Q^617^ cannot recognize the other CG pair in a CpG site. Comparing with other CXXC family members, the loss of hydrogen bonds with S^615^ and Q^617^ in TET1 CXXC is presumably responsible for binding with CpH DNA (H for A, T, or C) (22). In addition, the backbone of TET1 CXXC is more flexible compared with other CXXC family members, possibly resulting in binding promiscuity by accommodating any base pair following the CG pair in a CpH motif. A CCGG motif contains both CpC and CpG for TET1 CXXC binding. Broad binding selectivity of TET1 CXXC on CpH may thus facilitate the recruitment of the epigenetic enzyme to regions with rich mCpG sites and result in demethylation of the mCpG target with the enzyme’s biochemical function.

Methylation of CCGG has little effect on the dissociation time of TET1 CXXC upon DNA-strand separation. We analyzed the site-specific pausing time of TET1 CXXC on the mCCGG hairpin (Fig. 4*D*). A single-exponential function can explain all the histograms well except that at the fully methylated mCCGG3 ($\begin{array}{l} mCmCGG\\ GGmCmC \end{array}$) (Fig. 4*E*). The pausing time distribution at mCCGG3 requires a double-exponential function, providing a short pausing time of 18 ms and a long one of 63 ms at F_test_ = 14.75 pN. The short pausing time of 18 ms is at the same level as that at mCCGG4-8 and agrees well to its neighboring CCGG site (24 ms) (Fig. 4*E*, *bottom*). Also, the short pausing time of 18 ms from the mCCGG hairpin is almost identical to that happens on the CCGG hairpin (t_S_ = ∼ 14 ms), suggesting that the six methylation patterns in the mCCGG hairpin do not affect the dissociation of TET1 CXXC from its binding motifs. The pausing time at the first unmethylated CCGG site in the mCCGG hairpin is 174 ms, close to the long pausing time of t_L_ = ∼ 140 ms revealed from the CCGG hairpin (Fig. 4*E*, *bottom*). The long pausing time at mCCGG3 is 63 ms, 3x less than that at mCCGG1 or that from the CCGG hairpin (Fig. 4*E*, *bottom*). The decreased time indicates that the methylation pattern of mCCGG3 ($\begin{array}{l} mCmCGG\\ GGmCmC \end{array}$) affects the complex dissociation event, although mCCGG3 does not change the dissociation time of TET1 CXXC from a single fully methylated CCGG site.

### Single-molecule multiplexed profiling of a protein on a natural CGI sequence

**Figure 5:**
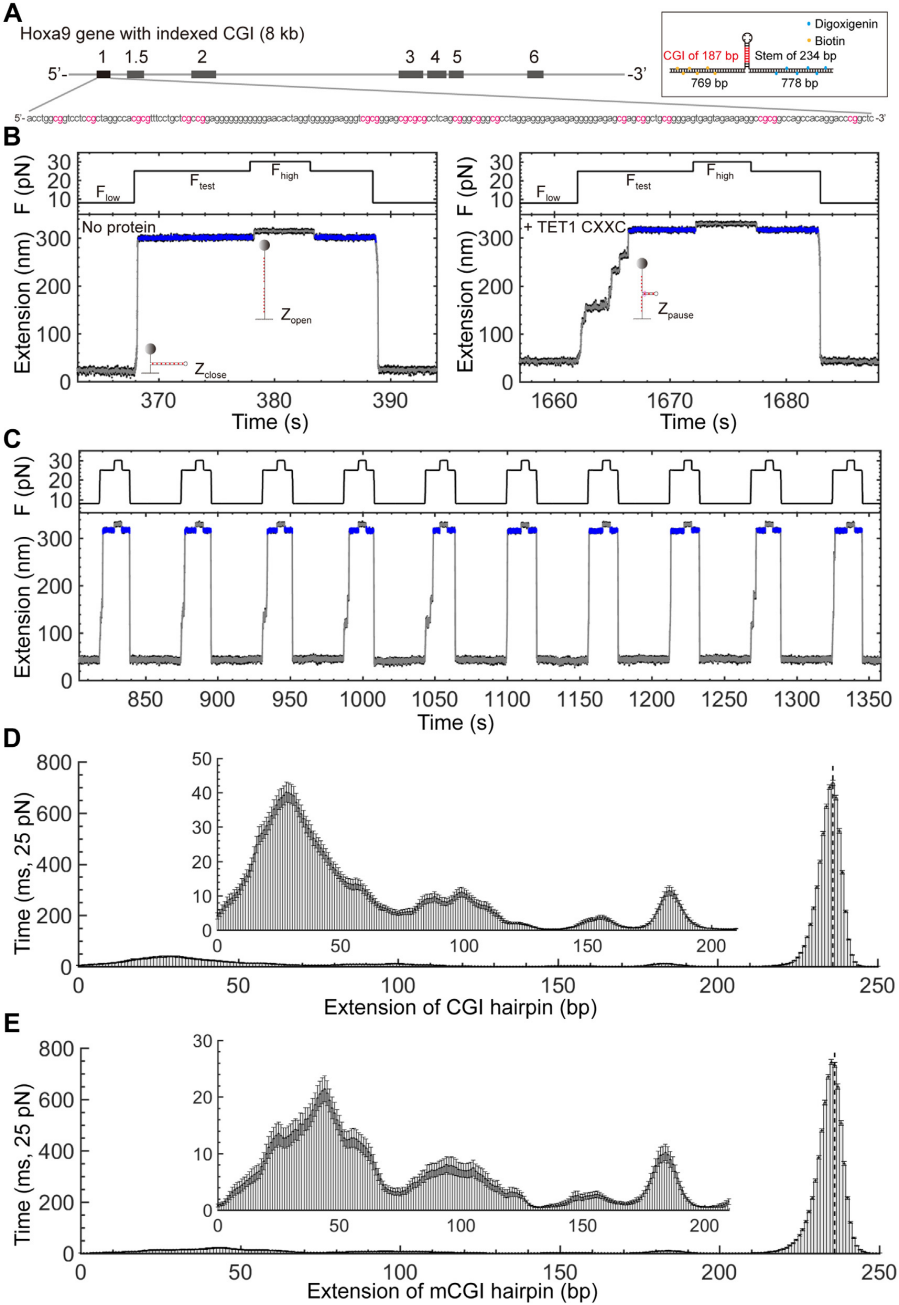
**Single-molecule multiplexed profiling of TET1 CXXC binding the CGI/mCGI hairpins.***A*, The CGI sequence from the mouse hoxa9 gene. CpGs are highlighted in *red*. Inset shows the hairpin construct with the CGI sequence. *B*, Single-molecule profiling assays with (*Right*) or without (*Left*) TET1 CXXC. [TET1 CXXC] = 0.25 μM. Buffer contains 10 mM of Tris (pH 7.4), 1 mM of EDTA, 100 mM of NaCl, 0.003% Tween-20, and 5 mM of DTT. Sampling rate = 400 Hz. *C*, Repetitive assays to examine the binding of TET1 CXXC on the CGI hairpin. *D*, Histograms of 2709 force-jumping traces for TET1 CXXC binding the CGI hairpin. Bin size = 2.46 bp. Height is converted to time by dividing the sampling rate of 400 Hz (Mean ± se). The *dotted line* indicates the hairpin loop position. A zoomed-up inset shows details. *E*, Histograms of 1890 force-jumping traces for TET1 CXXC binding the methylated mCGI hairpin. Same style and conditions as that in (*D*).

To examine TET1 CXXC-binding dynamics in a genome sequence background with crowed targets, we designed a DNA hairpin containing the first CpG island (CGI) from the promoter of the mouse Hoxa9 gene. The CGI of 187 bp (GC% = 73.8%) consists of 20 CpGs and 45 CpHs (H = A, T, or C), ∼ 8x more binding targets of TET1 CXXC than that in the previous CpG hairpins (eight binding motifs in 194 bp stem) (Fig. 5*A*) (26). The CGI is embedded in the hairpin stem of 236 bp, which introduces 20 more CpHs but none of CpG (Experimental procedures, Table S1). We find the critical force to be 20 ± 2 pN (Mean ± sd, n = 180) for entirely unfolding the CGI hairpin using force ramp assays at a force loading rate of 3 pN/s (Fig. S8). The GC-rich hairpin for the CGI (GC% = 67%) shows a much higher unfolding force than that of AT-rich hairpins (GC% < 16 %), *i.e.*, 20 pN for the CGI hairpin versus 12 pN for the CCGG hairpin, the ATCG hairpin, or the mCCGG hairpin. Using the force protocol with F_low_ = 8 pN, F_test_ = 25 pN, and F_high_ = 30 pN, we probe the footprints of TET1 CXXC on the CGI (Fig. 5*B*). The mechanical repetition allows us to statistically examine the TET1 CXXC binding the CGI upon forces jump from F_low_ to F_test_ (Fig. 5*C*). To reveal protein-binding behavior on DNA with dense CpG and CpH sites, we perform single-molecule multiplexed profiling assays on the CGI hairpin at a low concentration of TET1 CXXC (0.25 μM), about 12x less than its K_d_ (3.2 μM).

Crowded CpG and CpH motifs in the CGI hairpin blur the site-specific binding events by TET1 CXXC. We take 2709 traces to build the histograms for the nonmethylated CGI hairpin (Fig. 5*D*). Heights indicate the overall time of TET1 CXXC on the hairpin stem, at the same level as that happens at the downstream ATCG and mCCGG hairpins. The peaks are generally not in a well-defined Gaussian shape, and the baseline among peaks is high, suggesting that multiple binding events occur in proximity due to crowded CpG and CpH targets.

Methylated CGI hairpin shows a similar blurring phenomenon as that on nonmethylated CGI. We use the CpG methyltransferase to methylate CpGs of the CGI DNA to be mCpGs, which are validated by bisulfite sequencing (Experimental procedures). We construct the hairpin containing the CGI with mCpGs (mCGI) and run mechanical assays for TET1 CXXC under the same conditions as that of nonmethylated CGI. Using the methylated CGI hairpin, we build the histogram from 1890 force-jump traces (Fig. 5*E*). The histogram shows a noticeable blurring effect with multiple broad peaks and a high baseline at similar locations as that in the nonmethylated CGI hairpin.

**Figure 6:**
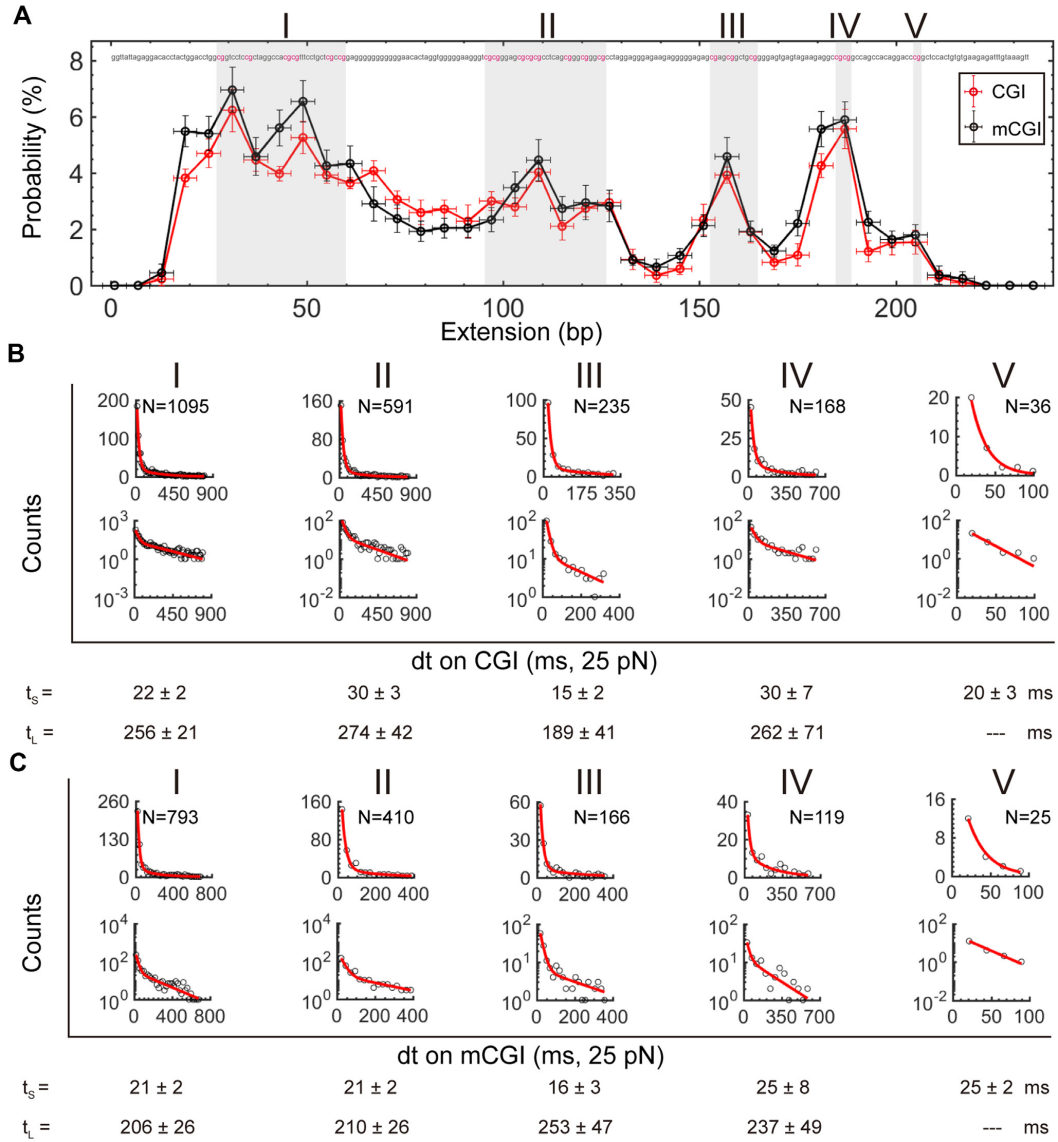
**Binding probabilities and dissociation time of TET1 CXXC on the CGI and mCGI hairpins.***A*, Averaged binding probabilities of TET1 CXXC along the CGI hairpin (*Red*, n = 3899 traces) and the mCGI hairpin (*Black*, n = 2445 traces). *Gray shadows* indicate the five CpG clusters (I-V) in the CGI. *Error bars* on y represent standard deviations. *Error bars* on x indicate a bin size of 6 bp. The CGI sequence is noted with CpGs in *red*. *B*, Dissociation time analysis for the five CpG clusters (I-V) in the nonmethylated CGI sequence. *Red curves* indicate a single- or double-exponential fitting. Fitting results are given with standard errors (Detailed fitting results in Table S3). *C*, Dissociation time analysis for CpG clusters I–V in the methylated mCGI sequence. Same style and data analysis as that in (*B*).

After measuring 3899 force-jumping traces for nonmethylated CGI, we can quantitatively examine the site-specific binding probabilities of TET1 CXXC on the CGI hairpin. Because TET1 CXXC concentration is low, we observe no bimodal effect on the binding probabilities along with the CGI sequence, unlike that on a CCGG hairpin (Fig. 6*A*, *red*). Five CpG clusters are evident from high binding probabilities in nonoverlapping bins of 6 bps (Fig. 6*A*, shadows). Clusters I, II, III, IV, and V, named according to the promoter direction from upstream to downstream, contain 6, 8, 3, 2, and 1 CpGs with CXXCs binding probabilities > 5x higher than that of base level. The clusters III, IV, and V are distinguished by sharp peaks of binding probabilities. In contrast, TET1 CXXC shows high plateaus of binding probabilities crossing multiple CpGs in clusters I and II. In clusters I and II, there are tandem CpGs, either (CpG)_2_ or (CpG)_3_. Also, clusters I and II have 10 and 3 CpH sites, respectively. With more targeting motifs than that in clusters III, IV, and V, multivalent interactions with TET1 CXXC may happen in clusters I and II, resulting in plateaus of binding probabilities.

To analyze site-specific binding probabilities on the mCGI hairpin, we collected 2445 traces (42 molecules) under the same experimental conditions as that for nonmethylated CGI hairpin. Methylation on CpGs does not change TET1 CXXC’s pattern of binding probabilities on the CGI (Fig. 6*A*, *black*). The site-specific difference of binding probabilities on CGI and mCGI is marginal at the low concentration of TET1 CXXC (0.25 μM).

We estimated the energy landscape from binding probabilities. Assuming a binding site with K_d_ in the CGI, at the concentration c of TET1 CXXC, we write the binding probability $P=c/\left(c+K_{d}\right)$. The ratio of the binding probabilities at the two sites will be: $\frac{P_{i}}{P_{j}}=\frac{c+K_{d}^{j}}{c+K_{d}^{i}}$, which depends on the concentration of TET1 CXXC (27). At a low concentration where c << $K_{d}^{i}$ and c << $K_{d}^{j}$, the ratio approaches $\frac{K_{d}^{j}}{K_{d}^{i}}$. Because [TET1 CXXC] of 0.25 μM << K_d_ of 3.2 μM, the site-specific binding probabilities reflect the relative binding affinities of TET1 CXXC along the CGI. Given the binding energy $\Delta G=-k_{B}Tln\left(P_{on}/P_{off}\right)$, where *k*_*B*_ is Boltzmann constant and P stands for probabilities of binding (on) and unbinding (off), we estimate the binding energies of TET1 CXXC on nonmethylated and methylated CGI (Fig. S9*A*) (18, 27, 28). Binding energies of TET1 CXXC at CpG clusters form visible valleys in the landscape along the CGI, while energy cost peaks at the nonCpG spacers (Fig. S9*A*, red). Energy valleys are deep and distinct at clusters III and IV. Meanwhile, valleys at clusters I and II are broad and rugged. The valleys in the energy landscape at the target CpG clusters can significantly facilitate TET1 CXXC to find their binding motifs when sliding in their proximity. CpG methylation has little effect on the shape of the energy landscape (Fig. S9*A*, black).

We further inspected the site-specific pausing time for TET1 CXXC to dissociate from the CGI or mCGI at F_test_. Under our testing conditions, the median of pausing time is generally long at CpG clusters and short at spacers between two clusters (Fig. S9*B*). CpG methylation does not change the overall pattern of TET1 CXXC’s pausing time along the CGI (Fig. S9*C*), which agrees well with the observation in the mCCGG hairpin. We collect the dissociation time from each CpG cluster and build histograms (Fig. 6*B* for CGI and 6c for mCGI). We find that the distributions of dissociation time for the five CpG clusters can be well fitted by a single or a minimum of double-exponential function. The resulted pausing time from a single-exponential function or the short pausing time from a double-exponential function is 15 ∼ 30 ms, which is at the same level as TET1 CXXC dissociation from a CpG site (Fig. 6*B*, bottom). In particular, cluster V contains a single CpG site and has a time distribution fitted by a single-exponential function as expected. Such agreement suggests that we have detected single events of TET1 CXXC dissociating from a target motif in a crowded context of CpGs and CpHs at a low concentration of 0.25 μM. The long pausing time from a double-exponential function is >200 ms with relatively large standard errors. Because of the disappearance of a bimodal phenomenon in binding probabilities at a low concentration of [TET1 CXXC] = 0.25 μM, the pausing time of >200 ms can be explained by the crowed targeting motifs in the CGI. Crowded binding motifs recruit multiple TET1 CXXC that could collaboratively resist the fork movement crossing clusters I–IV upon DNA-strand separation, making the dissociation time longer than that at cluster V with a single CpG site. Methylated mCGI reveals similar short and long pausing times at clusters I–V as that of nonmethylated CGI (Fig. 6*C*). The fact that TET1 CXXC’s dissociation from its targeting motifs in a CGI sequence is independent of CpG methylation agrees with the results from mCCGG hairpin.

## Discussion

Using single-molecule mechanical method, we here designed hairpin constructs of ∼200 bp DNA to quantitatively inspect how the TET1 CXXC domain dynamically interacts with CpGs in multiplexed sequences. By a measure of dissociation time, we reveal that strand separation forced TET1 CXXC to leave from a single binding target in tens of milliseconds. More interestingly, the dissociation time can be 10x longer at the fork of DNA-strand separation than that at the downstream DNA. In addition, the binding probability is high at the upstream DNA and low at the downstream DNA, independent from CpG’s flanking nucleotides or methylation patterns of cytosines.

The bimodal effects along the DNA strand separation direction may be explained by several possible mechanisms. One possibility comes from the nonspecific binding of protein on DNA. Sequence-specific DNA binding proteins interact with their targets by a rate exceeding the diffusion-limited on rate, *i.e.*, a protein can bind DNA nonspecifically (29). Because nonspecifically bound protein can slide on DNA (15), fork handles of dsDNA for mechanical manipulation could attract proteins and provide a pool of TET1 CXXC, which locally increases the binding probability at nearby CpG sites in the hairpin stem. TET1 CXXC bound on the upstream CpG sites close to the fork may also simultaneously interact with the nearby dsDNA handles nonspecifically, forming high-order protein–DNA complexes. This mechanism is consistent with the results that TET1 CXXC bound on the upstream hairpin has a longer dissociation time than that on the downstream hairpin. Also, the hypothesis of a high-order protein–DNA complex provides an explanation of why the dissociation time on the upstream hairpin cannot be fitted with a simple exponential function.

To serve as a general method for detecting protein–DNA interactions, we can minimize the nonspecific binding effect by either varying protein concentrations or simply ignoring the CpG sites close to the fork handles. The redundancy design of multiple CpG sites in a hairpin allows us to evaluate the sites beyond the influence of DNA handles.

When binding a single CpG site, the immediate flanking nucleotides have a dramatic effect on TET1 CXXC’s binding activity. By a measure of binding probability, G/C flanked CpG (GCGC) attracts TET1 CXXC 3x more often than that by C/G and A/T flanked ones, possibly because of the stacking forces along the DNA helical axis (30, 31). Since the binding probability approaches zero at T/A flanked CpG in our single-molecule assays, the difference is even more apparent comparing G/C and T/A flanking sequences. This finding is further validated by a measure of dissociation time at a force of ∼15 pN. The dissociation time of TET1 CXXC from a G/C flanked CpG is 4x slower than that from C/G or A/T flanked ones. A previous study using isothermal titration calorimetry showed similar K_d_ for TET1 CXXC binding C/G (3.1 μM), G/C (3.8 μM), A/T (2.4 μM), and T/A (3.5 μM) flanked CpGs (22). Our results demonstrated that single-molecule multiplexed profiling assays could provide alternative measurements other than K_d_, revealing distinguishable parameters to understand biomolecular interactions.

The single-molecule mechanical methods can break protein–DNA interacting sites one by one, which has been shown in numerous studies (16, 32, 33, 34, 35). Our single-molecule multiplexed profiling method unzips the dsDNA along the helical axis to detect the interactions between TET1 CXXC and DNA bases. The family of CXXC domains generally forms more and stronger hydrogen bonds with the CGs flanking a CpG site than the flanking ATs (22, 36). Therefore, the differential preferences on GC and AT flanked CpG sites indicate that we have detected the specific interactions between a CXXC protein and the flanking base pairs around a CpG site. Together, our findings of TET1 CXXC’s differential preference on the immediate neighboring bases around a CpG site are consistent with that in the literature (4) and further reveal the behavior of TET1 CXXC at the single-molecular level.

On a CGI sequence from the promoter of the mouse Hoxa9 gene, we measured the binding landscape of TET1 CXXC. Using ChIP-seq and direct bisulfite sequencing to compare the methylation patterns in Mll1^+/+^ and Mll1^-/-^ MEF cells, a previous study showed that MLL1 of a CXXC family protein protects five CpG clusters from methylation in the same Hoxa9 sequence as that we used (26). We find that the exact same five CpG clusters are clearly seen from the binding probabilities along the CGI sequence using our single-molecule multiplexed profiling method. The agreement between our *in vitro* and the previous *in vivo* results assures that our measurements of TET1 CXXC are accurate on a crowded target background with a great precision comparable with DNA sequencing.

To further investigate the dynamics of CXXC-DNA interactions, we can adjust experimental conditions using the single-molecule multiplexed profiling method. For example, by measuring the site-specific fraction of CXXC bound to DNA as a function of CXXC concentration, we can estimate the site-specific dissociation constants using the Hill equation (17). Note that the time of a protein on its DNA binding sites measured using our method is due to forced dissociation in a scenario of DNA strand separation, unlike that, a protein freely diffusing away from DNA. Experiments with a set of the enzyme concentrations may also clarify the nonuniform occupancy results along the DNA-strand separation direction. In addition, such experiments will allow us to directly measure the site-specific binding cooperativity of CXXCs to CpG and CpH clusters. Although we have tested at force = 14.75 and 25 pN here, we can measure binding energies as a function of testing forces according to Kramer’s Bell–Evans theory of force-dependent dissociation (18). By doing so, we should be able to determine site-specific kinetics and thermodynamics of interactions between a CXXC and DNA at force = 0 pN, *i.e.*, free of forces. We have examined the CXXC-binding event at a time interval of <60 s between two adjacent force jumps. We can systematically vary the incubation time to examine the CXXC-binding events at either equilibrated or nonequilibrated conditions (14).

In summary, we have developed a single-molecule approach that allows multiplexed profiling of protein–DNA complexes using magnetic tweezers. Our results demonstrate that this approach is feasible for studying the interactions between a protein and DNA with diverse binding motifs.

## Experimental procedures

Other than expressly noted, we have purchased all the chemicals from Sigma-Aldrich, the DNA oligos from Sangon Biotech, and the enzymes from New England Biolabs.

### Surface plasmon resonance

On a sensor chip (CM5, GE), we activate the matrix of carboxymethylated dextran using freshly prepared 70 μl of 0.4 M EDC (1-ethyl-3-(3-dimethylaminopropyl) carbodiimide, GE) and 0.1 M NHS (N-hydroxysuccinimide, GE) in an SPR instrument (Biacore T200, GE). The flow rate is 30 μl/min. After washing using 1 M ethanolamine-HCl (pH 8.5), we next flow streptavidin (10 μg/ml) to the dextran maxtrix, allowing immobilization reactions between the primary amine groups and the reactive succinimide esters *via* amine coupling for 7 min in a buffer containing 10 mM NaOAc (pH 5.0). We then wash the surface using ethanolamine to deactivate excess reactive groups. To capture the DNA ligand by streptavidin, we continue to load 1 μg/ml of biotin modified CCGG dsDNA (Biotin-GCCAACCGGAACCG in 10 mM Tris (pH 7.4)) onto the chip until reaching an immobilized level of ∼250 RU (SPR response units). We wash the extra DNA away using a running buffer containing 10 mM HEPES (pH 7.4), 150 mM NaCl, 3 mM EDTA, and 0.005% Tween 20. On the SPR chip with biotin-modified CCGG DNA, we finally titrate TET1 CXXC at concentrations of 0.5 μM, 1 μM, 2 μM, 4 μM, and 8 μM in a buffer containing 10 mM Tris (pH 7.4), 100 mM NaCl, 50 μM ZnCl_2_, 1 mM DTT, 3% glycerol, and 0.005% Tween 20. After each measurement at a concentration of TET1 CXXC, we regenerate the SPR surface using 0.5% SDS.

To analyze the data, we first subtract a blank sensorgram from the ones with TET1 CXXC. The adjusted sensorgrams are fitted to a kinetics model using the evaluation software of the SPR instrument (Biacore T200, GE). The estimated k_on_, k_off_, and K_d_ are reported.

### DNA constructs

Our hairpin constructs, CCGG hairpin, ATCG hairpin, mCCGG hairpin, and CGI hairpin, consist of parts for mechanical manipulation and unique sequences of interest. The general parts for mechanical manipulation are two handles, a junction, and a loop. To make handles for affinity interactions, we run PCR to prepare DNA fragments of 676 bp using a template of pBluescript II SK(+) (Cat#: 212205, Agilent, USA) and a dNTP mixture supplemented with biotin-16-dUTP or digoxigenin-11-dUTP (Cat#: 11093070910 or 11093088910, Roche, Switzerland) (Forward and reverse primers in Table S1). Restriction sites of BbvCI or PpuMI serve for ligation between two handles and a hairpin junction, which is assembled from four DNA oligos (Junction oligos in Table S1). For CCGG, mCCGG, and ATCG hairpins, we use two DNA linkers to connect the junction and handles due to the conflict of restriction sites (Linker oligos in Table S1). Sequences of interest in the hairpin stem between a junction and a loop are made of either synthesized oligos for CCGG/mCCGG/ATCG hairpins or PCR products (Stem oligos and loop oligos in Table S1). For ligating the stems of CCGG/mCCGG/ATCG hairpins with or without methylated cytosines using T4 ligase, we mix the stem oligos, which are phosphorylated using T4 polynucleotide kinase and heated up to 95 °C for 5 min, followed by slowly cooling down to room temperature in a few hours. We use PCR to generate the stem sequence in the CGI hairpin (Primer oligos in Table S1). We use M.SssI to methylate all CpGs in the stem of a CGI hairpin at 37 °C for 4 h in a reaction buffer coming together with methyltransferase (Cat#: M0226S, NEB, USA). The DNA methylation in the CGI hairpin is validated by bisulfite sequencing (Servicebio, China).

### Magnetic tweezers

We use home-made magnetic tweezers (Fig. S1), similar to the setup previously described (37, 38, 39, 40), which consists of microscopy, motors manipulating magnets, Piezo stages, and a flow cell connecting to a pump. The inverted microscope uses an oil immersion objective (UPLFLN 100 × O2, Numerical aperture (N.A.) = 1.3; Olympus, Japan) and a tube lens (Cat#: AC508–400-A, f = 400 mm, Thorlabs, USA) to achieve a magnification of 222×. A CMOS camera (Cat#: MC1362, Mikrotron, Germany) serves for imaging. The frame grabber device of PCIe 1433 (National Instruments, USA) receives images through Camera Link cables. A CUDA supporting graphics card of GeForce GTX 1060 (NVIDIA, USA) and a CPU of Intel Core i7-6700 (Intel, USA) further handle the images in a desktop computer supported by Windows 10 (64 bit). A pair of NdFeB magnets (Cat#: W-05-N50-G, 5 mm cube, Webcraft GmBH, Germany) is vertically aligned and separated by 1 mm gap, which allows illumination from a 660 nm LED (Cat#: M660F1, Thorlabs). A translate stage (Cat#: M-404.1PD, Physik Instrumente, Germany) controls the magnets in the z-direction. A piezo nanopositioner (Cat#: P-726.1CD, Physik Instrumente) moves the objective in the z-direction. We use two coverslips (Cat#: S1699–12–100EA, i-Quip, USA) to make a flow cell with a single channel that is shaped by double layers of parafilm and connected to a peristaltic pump (Cat#: ISM832, Ismatec, Germany).

A custom-written application in Labview 2017 serves as a user interface for hardware configurations, experimental measurements, and data analysis in magnetic tweezers, similar to that published in literature (41). In this application, we use a Quadrant Interpolation algorithm for accurately and simultaneously analyzing video-based images of microspheres. In a CUDA parallel computing framework, we generally track microspheres and obtained their XYZ coordinates in an ROI of 150 pixels at a sampling rate of >200 Hz.

### Single-molecule assays using magnetic tweezers

We run single-molecule assays using a setup of microsphere-DNA-coverslip in a microfluidic chamber. We routinely mix 1 ng of DNA hairpins with 20 μl of streptavidin-coated microspheres (Cat#: 65,305, M270, Invitrogen, USA) in a buffer of 40 μl containing 10 mM of Tris (pH 7.4), 1 mM of EDTA, 100 mM of NaCl and 0.003% Tween-20. We make a matrix of nitrocellulose (0.1%, m/v) on a coverslip, which is later incubated with anti-digoxigenin antibody (0.1 mg/ml, Cat#: 11093274910, Roche) for 2–4 h and passivated with BSA (5 mg/ml) for overnight. Coverslip surface with an antibody can immobilize the DNA molecules that are already bound to microspheres. We run both force-ramp and force-jump assays in a buffer containing 10 mM of Tris (pH 7.4), 1 mM of EDTA, 100 mM of NaCl, 0.003% Tween-20 and 5 mM of DTT. We purchased TET1 CXXC from Sangon Biotech Co, Ltd (Shanghai, China) (Table S2). The tested concentration of TET1 CXXC should be high enough to give detectable signals in a reasonable timescale, while the concentration should also be low enough to avoid nonspecific binding between magnetic beads and glass surface.

Mechanical profiling of TET1 CXXC on DNA is based on a force protocol in force-jump assays with a sampling rate of >200 Hz. At a low force (F_low_), which is less than the critical force of unfolding a hairpin, the DNA hairpin stays closed at short extensions. In 390 ± 30 milliseconds (mean ± sd, n = 10), we quickly increase the force from F_low_, crossing the critical force, to a testing force (F_test_) where the hairpin is fully opened at long extensions. The opening of a hairpin can be blocked by CXXC-binding events, which will produce pausing signals at intermediate extensions. We continue to increase the force from F_test_ to a higher force (F_high_) where the protein–DNA complex should fully dissociate, and the DNA further extends (18, 42). We then decrease the F_high_ to F_test_, which serves for reference purposes. We finally lower down the F_test_ to F_low_, which completes a circle of our force protocol. We can tune the F_low_, F_test_, and F_high_, as well as their durations, of a force protocol according to the tested proteins and hairpin constructs, a method similar to that published (14, 16, 17, 18,43, 44).

### Data analysis for single-molecule profiling assays

We have analyzed all the single-molecule data in MatLab (R2017a, Mathworks, USA). We directly measure the pausing time in traces of force-jump assays. Because data are collected at a minimum of 200 Hz, we only collect the pausing time >10 ms considering the Nyquist frequency, *i.e.*, pausing events with dwell times longer than 10 ms would qualify as “pauses”. A protein-binding event at a CpG site is determined to be observed within ±11 bp around the CpG site for the CCGG/XCGY/CmCGG hairpins. To reduce the effect of outliers in the heavy-tailed distributions upon fitting, we follow a binning rule of the Freedman–Diaconis algorithm to build time histograms. We use exponential functions to fit the probability distributions of pausing times, which are independent Poisson processes. For a single-exponential function, we use the equation of $f\left(x\right)=y_{0}.\exp\left(\frac{x}{\tau }\right)\;$. For two simultaneous independent Poisson processes with different rates, the distribution of the pausing times is simply a linear combination of the single-exponential functions, i.e., $f\left(x\right)=y_{1}.\exp\left(\frac{x}{\tau_{1}}\right)+\;y_{2}.\exp\left(\frac{x}{\tau_{2}}\right)$. Detailed fitting results with statistics are in Table S3.

To measure the pausing positions, we perform zero correction for each trace by subtracting the bead height where the hairpin fully opens (Z_open_) at the force of F_test_. After zero correction at F_test_, the resulting change in extensions represents the length of ssDNA released while unfolding a hairpin. We next build a histogram for the extension of each trace. The pausing positions at specific extension form histogram peaks that indicate the blockage of hairpin unfolding due to CXXC binding. We convert the pausing positions from nanometer to bp according to the CCGG hairpin. The CCGG construct contains eight CCGG sites, which are evenly distributed in the hairpin stem, forming eight featured peaks upon CXXC binding and serving as a ruler for the unit conversion.

To theoretically calculate the conversion factor of single nucleotide length based on force (F) as a function of extension (x), we use the Marko–Sigga formula of a worm-like chain model (45),

(Equation 1) $$F=\frac{k_{B}T}{L_{p}^{ss}}(\frac{1}{4}{\left(1-\frac{x}{L_{c}^{ss}}\right)}^{-2}+\frac{x}{L_{c}^{ss}}-\frac{1}{4})$$

where $L_{p}^{ss}$ stands for the persistence length of ssDNA, $L_{c}^{ss}$ for the contour length of ssDNA, *k*_*B*_ for the Boltzmann constant, and T for the temperature. We take the values of $L_{p}^{ss}$ = 0.87 nm of ssDNA and $L_{c}^{ss}$ = 0.69 nm for a single nucleotide from the experimentally determined ones by A. Bosco *et al*. at a salt condition with 100 mM NaCl (24).

## Data availability

All data referred to is included in this paper.

## Conflict of interests

The authors declare that they have no conflicts of interest with the contents of this article.
