# Supplementary material for: Single-molecule multiplexed profiling of protein–DNA complexes using magnetic tweezers
Source: J Biol Chem. 2021 Jan 23;296:100327. doi: 10.1016/j.jbc.2021.100327 (PMC7949110; doi:10.1016/j.jbc.2021.100327)
Supplement: Supplementary file 1 — Tables and Figures [file mmc1.pdf]

Supporting Information

Single-molecule Multiplexed Profiling of Protein–DNA Complexes Using Magnetic Tweezers

Lin Liang[1], Zeyu Wang[1], Lihua Qu[1], Wei Huang[1], Shuang Guo[1], Xiangchen Guan[1], Wei Zhang[2], Fuping Sun[2], Hongrui Yuan[2], Huiru Zou[3], Haitao Liu[2], Zhongbo Yu[1]*

[1] State Key Laboratory of Medicinal Chemical Biology, College of Pharmacy, Nankai University, 38 Tongyan Road, Tianjin 300350, China

[2] Tianjin Key Laboratory of Optoelectronic Sensor and Sensing Network Technology, Institute of Modern Optics, College of Electronic Information and Optical Engineering, Nankai University, Tianjin 300350, China

[3] Central Laboratory of Tianjin Stomatological Hospital, The Affiliated Stomatological Hospital of Nankai University, Tianjin 300041, China

* To whom correspondence should be addressed. Email: zyu@nankai.edu.cn

This Supplementary Information includes:

Table S1. Oligos for constructing DNA hairpins

Table S2. Sequences of TET1 CXXC

Table S3. Fitting results of pausing time distributions

Figure S1. Unfolding and refolding of a CCGG hairpin using single-molecule magnetic tweezers

Figure S2. Sensorgrams of SPR assays for the synthetic TET1 CXXC binding a dsDNA

Figure S3. Bimodal effect responded to the concentration of TET1 CXXC

Figure S4. Bimodal effect responded to the mutations of CCGG1-3

Figure S5. Bimodal effect responded to the pattern changes of CCGG density

Figure S6. ATCG hairpin shows no pauses upon unfolded by a testing force of 14.75 pN

Figure S7. Hairpin of mCCGG shows no pauses upon unfolded by a testing force of 14.75 pN

Figure S8. Unfolding and refolding of a CGI hairpin using single-molecule force spectroscopy.

Figure S9. Energy landscapes and dissociation time of TET1 CXXC on the CGI/mCGI hairpins.

Table S1. Oligos for constructing DNA hairpins.

Name	Sequence (5′-3′)
**Junction 1 for CCGG hairpin**	GAGATGATTTGAAAAAATATGAAGAATGGTATAATAAAAGGGTGATTTATATTTATTTATTCCGGTATTTAATTTAATTATATCCG
**Junction 2 for CCGG hairpin**	AATATATAACCGGATATAATTAAATTAAATACCGGAATAAATAAATATAAATCTGGGAGTAGATGTGGTTTTTGTTTGTTTG
**Junction 3 for CCGG hairpin and ATCG hairpin**	CCCTTTTATTATACCATTCTTCATATTTTTTC
**Junction 4 for CCGG hairpin and ATCG hairpin**	ACTCATCATTCAAACAAACAAAAACCACATCTACTCCC
**Stem 1 for CCGG hairpin with or without methylation**	GTTATATATTTATATTTAT/i5MedC//i5MedC/GGTTTATTTATTATTTATTT/i5MedC/CGGTTATTTATAATTTAATTAC
**Stem 1c for CCGG hairpin with or without methylation**	ATAATAAC/i5MedC/GGTAATTAAATTATAAATAA/i5MedC/CGGAAATAAATAATAAATAAA/i5MedC//i5MedC/GGATAAATATA
**Stem 2 for CCGG hairpin with or without methylation**	/i5MedC/GGTTATTATATATTATTTAT/i5MedC//i5MedC/GGTATTTATTTAATTATATT/i5MedC/CGGTTATTTATATATTTATATC/i5MedC/
**Stem 2c for CCGG hairpin with or without methylation**	ATATAAATAACCGGAATATAATTAAATAAATACCGGATAAATAATAT
**Loop for CCGG hairpin**	GGTTAATATTTATTATATTTTTTTAAATATAATAAATATTAACCGGATATAAAT
**Junction 1**	GAGATGATTTGAAAAAATATGAAGAATGGTATAATAAAA

**for dense CCGG hairpin**	GGgTGATTTATATTTATTTATTCCGGTATTTAACCGGTTTATTTCCG
**Junction 2 for dense CCGG hairpin**	GGTTTATAACCGGAAATAAACCGGTTAAATACCGGAATAAATAAATATAAATCTgGGAGTAGATGTGGTTTTTGTTTGTTTG
**Stem 1 for dense CCGG hairpin**	GTTATAAACCGGTATTTATCCGGTTAAATTCCGGTTTATTTCCGGTTATTTATAATTTAATTAC
**Stem 1c for dense CCGG hairpin**	ATAATAACCGGTAATTAAATTATAAATAACCGGAAATAAACCGGAATATAACCGGATAAATACC
**Junction 1 for CATG hairpin**	GAGATGATTTGAAAAAATATGAAGAATGGTATAATAAAAGGgTGATTTATATTTATTTATTCATGTATTTAATTTAATTATATCAT
**Junction 2 for CATG hairpin**	AATATATAACATGATATAATTAAATTAAATACATGAATAAATAAATATAAATCTgGGAGTAGATGTGGTTTTTGTTTGTTTG
**Stem 1 for CATG hairpin**	GTTATATATTTATATTTATCATGTTTATTTATTATTTATTTCCGGTTATTTATAATTTAATTAC
**Stem 1c for CATG hairpin**	ATAATAACCGGTAATTAAATTATAAATAACCGGAAATAAATAATAAATAAACATGATAAATATA
**Junction 1 for ATCG hairpin**	GAGATGATTTGAAAAAATATGAAGAATGGTATAATAAAAGGGTGATTTATATTTATTTATTCCGGTATTTAATTTAATTATATGCG
**Junction 2 for ATCG hairpin**	AATATATAAGCGCATATAATTAAATTAAATACCGGAATAAATAAATATAAATCTGGGAGTAGATGTGGTTTTTGTTTGTTTG
**Stem 1 for ATCG hairpin**	CTTATATATTTATATTTATACGTTTTATTTATTATTTATTTTCGATTATTTATAATTTAATTAC
**Stem 1c for ATCG hairpin**	ATAATAACCGGTAATTAAATTATAAATAATCGAAAATAAATAATAAATAAAACGTATAAATATA

**Stem 2 for ATCG hairpin**	CGGTTATTATATATTATTTATGCGCTATTTATTTAATTATAT TACGTTTATTTATATATTTATATTC
**Stem 2c for ATCG hairpin**	ATATAAATAAACGTAATATAATTAAATAAATAGCGCATAAA TAATAT
**Loop for ATCG hairpin**	GATTAATATTTATTATATTTTTTTAAATATAATAAATATTAAT CGAATATAAAT
**Linker 1 for CCGG hairpin and ATCG hairpin**	TCAGCAAGGAAGGAGATTTTGAAAAATTTATTTATTAGA TATTGGAAATATTATTAGAG
**Linker 1c for CCGG hairpin and ATCG hairpin**	AAATCATCTCCTCTAATAATATTTCCAATATCTAATAAATA AATTTTTCAAAATCTCCTTCCTTGC
**Linker 2 for CCGG hairpin and ATCG hairpin**	AATGATGAGTGTTAAAAAAAGTGGGGAAGTGAGTAATG AAATTATTTTGTATGTTTTTTATATGAATTTATTTTTTGGG
**Linker 2c for CCGG hairpin and ATCG hairpin**	GACCCCAAAAAATAAATTCATATAAAAAACATACAAAATA ATTTCATTACTCACTTCCCCACTTTTTTTAAC
**Junction 1 for CGI hairpin**	TCAGCAAGGAAGGAGATTTTGAAAAATTTATTTATTAGA TATTGGAAATATTATTAGAGGAGATGATTTAAAAAAATAT GAAGAATGGTATAATAAAAGGGTTTGGTTATTAGAGGA CACCTA
**Junction 2 for CGI hairpin**	GTGTCCTCTAATAACCTTTGGGAGTAGATGTGGTTTTT GTTTTTTTGAATAATAAATGTTAAAAAAAGTGGGGAAGT GAGTAATGAAATTATTTTGTATGTTTTTTATATGAATTTAT TTTTTGGG
**Junction 3 for CGI hairpin**	CCCTTTTATTATACCATTCTTCATATTTTTTTAAATCATCT CCTCTAATAATATTTCCAATATCTAATAAATAAATTTTTCA AAATCTCCTTCCTTGC

**Junction 4 for CGI hairpin**	GACCCCAAAAAATAAATTCATATAAAAAACATACAAAATAATTTCATTACTCACTTCCCCACTTTTTTTAACATTTATTATTCAAAAAAACAAAAACCACATCTACTCCC
**Loop for CGI hairpin**	GTGAAGAGATTTGTAAAGTTTTCTTTACAAATCTCTTCACACA
**Sequence of the CGI**	ACCTGGCGGTCCTCCGCTAGGCCACGCGTTTCCTGCTCGCCGGAGGGGGGGGGGGAACACTAGGTGGGGGAAGGGTCGCGGGAGCGCGCGCCTCAGCGGGCGGGCGCCTAGGAGGGAGAAGAGGGGGAGAGCGAGCGGCTGCGGGGAGTGAGTAGAAGAGGCCGCGGCCAGCCACAGGACCCGGCTC
**Forward primer for CGI sequence**	ATATTTCCACCTACTGGACCTGGC
**Reverse primer for CGI sequence**	ATATTTCCAGACAGTGGAGCCGG
**Forward primer for the biotin handle**	GACCGAGATAGGGTTGAGTG
**Reverse primer for the biotin handle**	GCATCGGCTGAGGAAAGGGAACAAAAGCTGG
**Forward primer for the digoxigenin handle**	ATCGTAGGGTCCTGACCGAGATAGGGTTGAGTG
**Reverse primer for the digoxigenin handle**	AAAGGGAACAAAAGCTGG

Notes: /i5MedC/ indicated a methylated cytosine if a methylated CCGG hairpin should be prepared.

Table S2. Sequences of TET1 CXXC.

Name	Sequences of amino acids	Length
TET1 CXXC	KRKRCGVCEPCQQKTNCGECTYCKNRKNSHQICKKR KCEELKKKPS	46

Notes: TET1 CXXC has 46 amino acids with a molecular weight of 5.4 kDa. The basic peptide carries 10.5 positive charges at pH = 7.4 with PI = 10.1. The lyophilized peptide is soluble in water. We prepared a stock solution with 0.7 mM of the peptide in a buffer containing 10 mM Tris (pH 7.4), 100 mM NaCl, 50 μM $ZnCl_2$, 1 mM DTT, and 30% glycerol. The stock solution can be further diluted with an assay buffer.

Table S3. Fitting results of pausing time distributions.

Hairpin constructs	Binding Sites	t (ms)	RMSE	R-Squared	p-value	Bin size (ms)	N
**CCGG hairpin**	CCGG1	126 ± 16	1.07	0.91	$9x10^{-5}$	49.25	164
	CCGG2	15 ± 4	1.03	0.902	$2x10^{-20}$	10.48	217
	CCGG2	149 ± 26					
	CCGG3	24 ± 14	0.801	0.979	$9x10^{-15}$	20.36	243
	CCGG3	142 ± 23					
	CCGG4	14 ± 1	0.961	0.965	$9x10^{-4}$	14	125
	CCGG5	10 ± 1	0.603	0.993	$2x10^{-4}$	7.71	57
	CCGG6	12 ± 1	0.553	0.904	$2x10^{-5}$	12.25	45
	CCGG7	9 ± 2	4.49	0.878	$6x10^{-4}$	11.75	44
	CCGG8	7 ± 1	0.928	0.991	$5x10^{-4}$	12.14	41
**ATCG hairpin**	CCGG	159 ± 4	3.49	0.991	$1x10^{-42}$	20.94	1064
	GCGC	63 ± 1	4.01	0.996	$1x10^{-25}$	18	1073
	ACGT	14 ± 1	3	0.989	$1x10^{-13}$	4.3	234
	TCGA	--	--	--	--	--	--
	CCGG	13 ± 1	1.99	0.989	$3x10^{-9}$	8	183
	GCGC	50 ± 3	1.07	0.986	$5x10^{-18}$	10.63	224
	ACGT	--	--	--	--	--	--
	TCGA	--	--	--	--	--	--
**mCCGG hairpin**	CCGG	174 ± 2	1.67	0.999	$3x10^{-30}$	54.8	2803
	CCGG	24 ± 0.5	5.6	0.998	$7x10^{-20}$	12.25	4350
	mCmCGG	18 ± 3	0.781	0.999	$9x10^{-27}$	10	2990
	mCmCGG	63 ± 4					
	mCCGG	17 ± 1	1.45	0.978	$1x10^{-11}$	4.56	663
	CmCGG	18 ± 1	1.33	0.98	$6x10^{-9}$	9.25	139
	mCmCGG	18 ± 1	1.44	0.977	$3x10^{-6}$	8.63	155

	mCCGG	9 ± 0.4	1.56	0.992	$1x10^{-7}$	5.67	129
	CmCGG	8 ± 0.3	0.846	0.997	$3x10^{-6}$	3.75	85
**CGI hairpin**	Cluster I	22 ± 2	0.846	0.93	$6x10^{-38}$	12.48	1095
	Cluster I	256 ± 21					
	Cluster II	30 ± 3	0.913	0.98	$1x10^{-31}$	19.53	591
	Cluster II	274 ± 42					
	Cluster III	15 ± 2	0.467	0.982	$1x10^{-9}$	22.86	235
	Cluster III	189 ± 41					
	Cluster IV	30 ± 7	0.71	0.901	$1x10^{-9}$	31.13	168
	Cluster IV	262 ± 71					
	Cluster V	20 ± 3	0.619	0.944	$6x10^{-3}$	19.88	36
**mCGI hairpin**	Cluster I	21 ± 2	1.06	0.938	$1x10^{-19}$	19.96	793
	Cluster I	206 ± 26					
	Cluster II	21 ± 2	0.964	0.975	$2x10^{-11}$	24.43	410
	Cluster II	210 ± 26					
	Cluster III	16 ± 3	0.88	0.909	$4x10^{-12}$	16.9	166
	Cluster III	253 ± 47					
	Cluster IV	25 ± 8	1.1	0.969	$1x10^{-9}$	42.85	119
	Cluster IV	237 ± 49					
	Cluster V	25 ± 2	0.334	0.997	$1x10^{-3}$	22.73	25

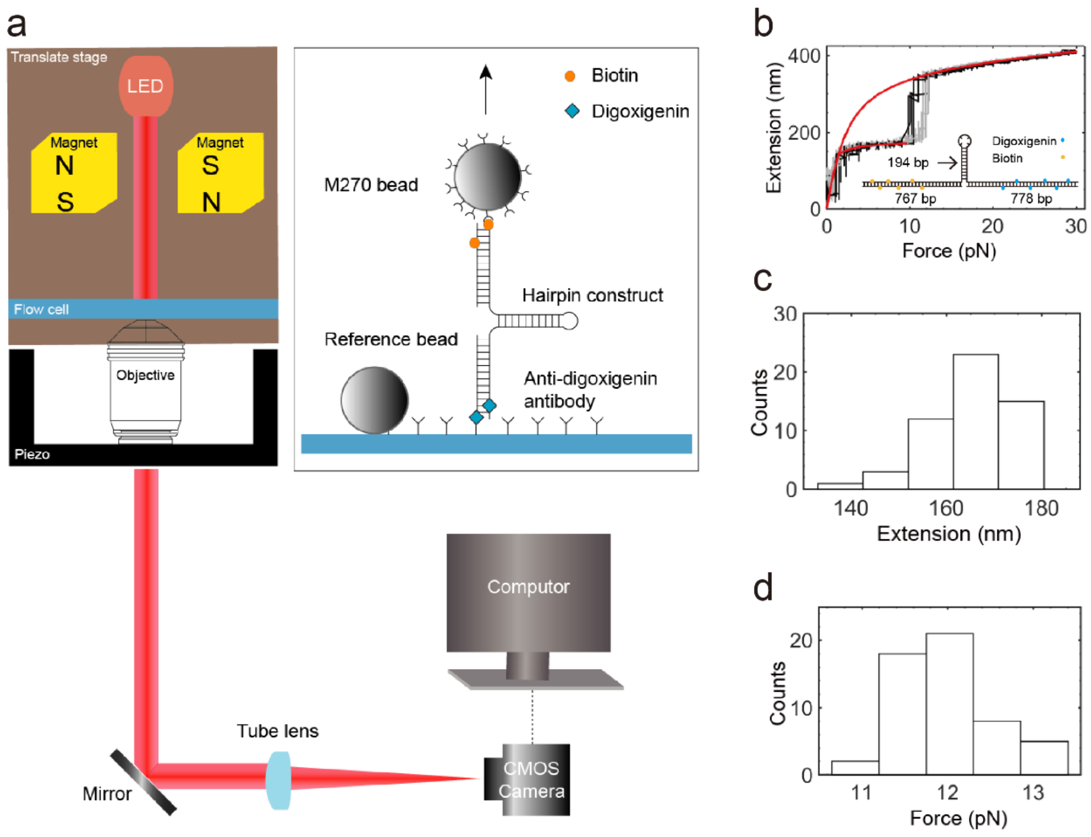

Figure S1. Unfolding and refolding of a CCGG hairpin using single-molecule magnetic tweezers

(a). Magnetic tweezers setup.

(b). Mechanical manipulation of the CCGG hairpin using single-molecule force spectroscopy. Inset shows the construct of the CCGG hairpin. The force loading rate is 3 pN/s. The stretching process is in gray color and the relaxing process in black. Red lines are worm-like-chain fittings.

(c). Histogram of changes in extension (N = 54).

(d). Histogram of unfolding forces. The averaged force is 11.9 ± 0.6 pN (mean ± sd, n = 54) regarding the entirely unfolded state.

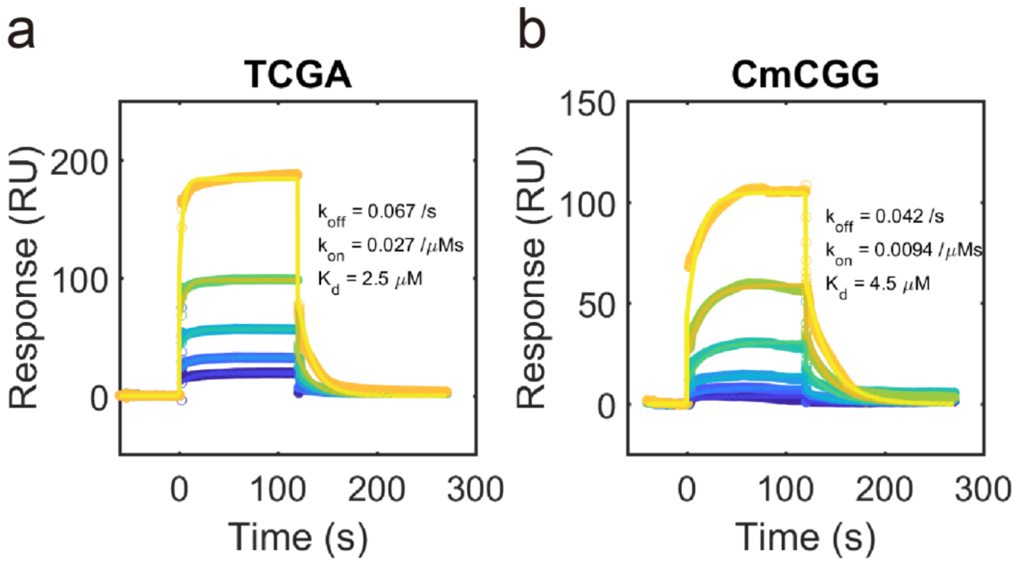

Figure S2. Sensorgrams of SPR assays for the synthetic TET1 CXXC binding a dsDNA

(a). SPR for TET1 CXXC binding a TCGA dsDNA. K_d = 2.5 μM. TET1 CXXC concentrations are 0.5 μM, 1 μM, 2 μM, 4 μM, and 8 for the TCGA dsDNA. The TCGA dsDNA is the same sequence as the CCGG dsDNA used in Figure 1b except that we replace the CCGG site with a TCGA site. The experimental conditions used for the TCGA dsDNA are the same as those for the CCGG dsDNA in Figure 1b.

(b). SPR for TET1 CXXC binding a CmCGG dsDNA. K_d = 4.5 μM. TET1 CXXC concentrations are 0.125 μM, 0.25 μM, 0.5 μM, 1 μM, 2 μM, and 4 μM for the CmCGG dsDNA. The CmCGG dsDNA is the same sequence as the CCGG dsDNA used in Figure 1b except that the central CpG site is fully methylated. We have used a PBS-based running buffer here. Other experimental conditions are the same as those for the CCGG dsDNA in Figure 1b.

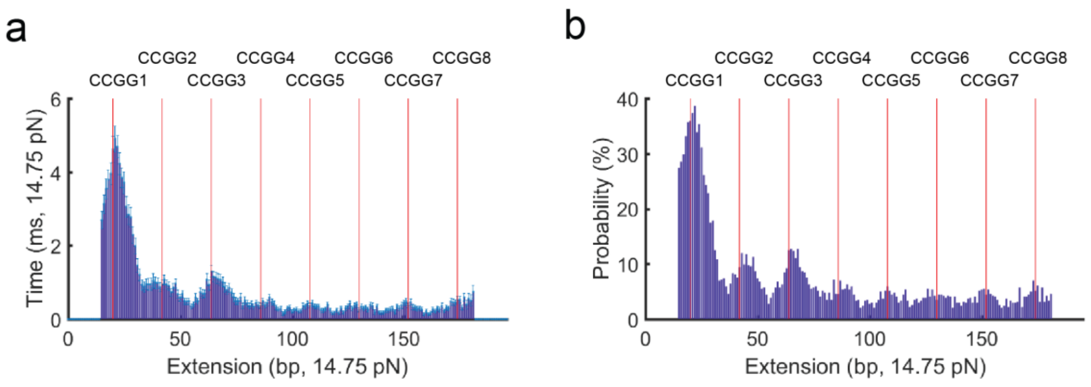

Figure S3. Bimodal effect responded to the concentration of TET1 CXXC

(a). Overall dwell time distribution on the CCGG hairpin with [TET1 CXXC] = 0.8 μM. Red vertical lines indicate the positions of CCGG sites. Error bars represent standard error. N = 398 traces from 19 molecules. Bin size = 1 bp. Other experimental conditions are the same as those in Figure 2a.

(b). Binding probability on the CCGG hairpin with [TET1 CXXC] = 0.8 μM. Data are derived from (a). Bin size = 1 bp.

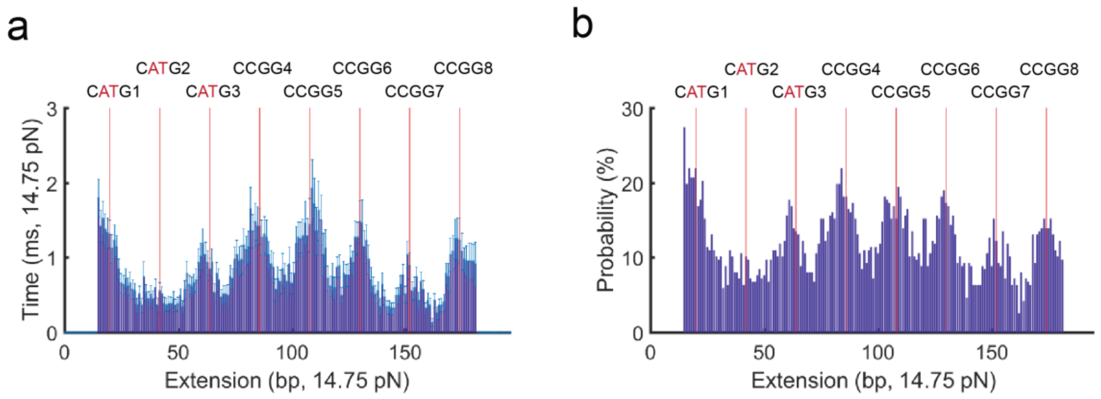

Figure S4. Bimodal effect responded to the mutations of CCGG1-3

(a). Overall dwell time distribution on the CATG hairpin. Red vertical lines indicate the positions of CATG and CCGG sites. Error bars represent standard error. N = 237 traces from 31 molecules. Bin size = 1 bp. Other experimental conditions are the same as those in Figure 2a.

(b). Binding probability on the CATG hairpin. Data are derived from (a). Bin size = 1 bp.

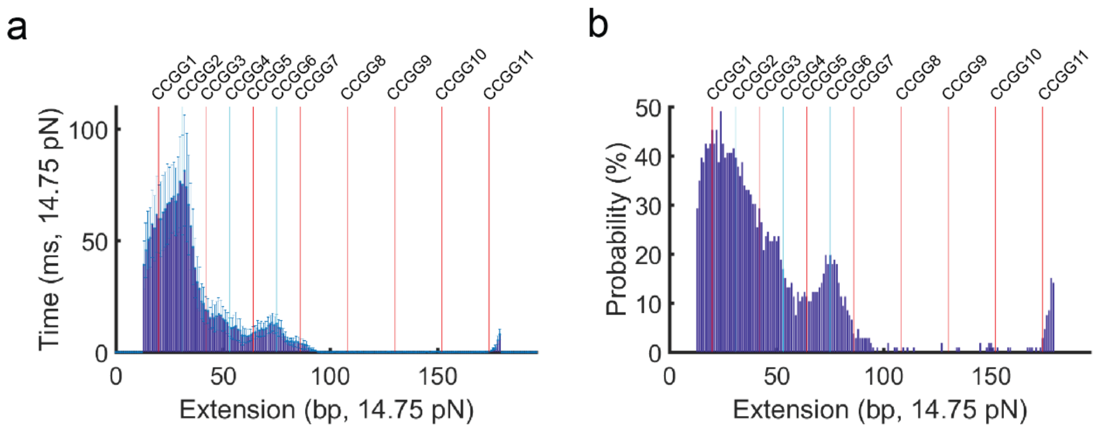

Figure S5. Bimodal effect responded to the pattern changes of CCGG density

(a). Overall dwell time distribution on the dense CCGG hairpin. Red and cyan vertical lines indicate the positions of CCGG sites. Error bars represent standard error. N = 106 traces from 12 molecules. Bin size = 1 bp. Other experimental conditions are the same as those in Figure 2a.

(b). Binding probability on the dense CCGG hairpin. Data are derived from (a). Bin size = 1 bp.

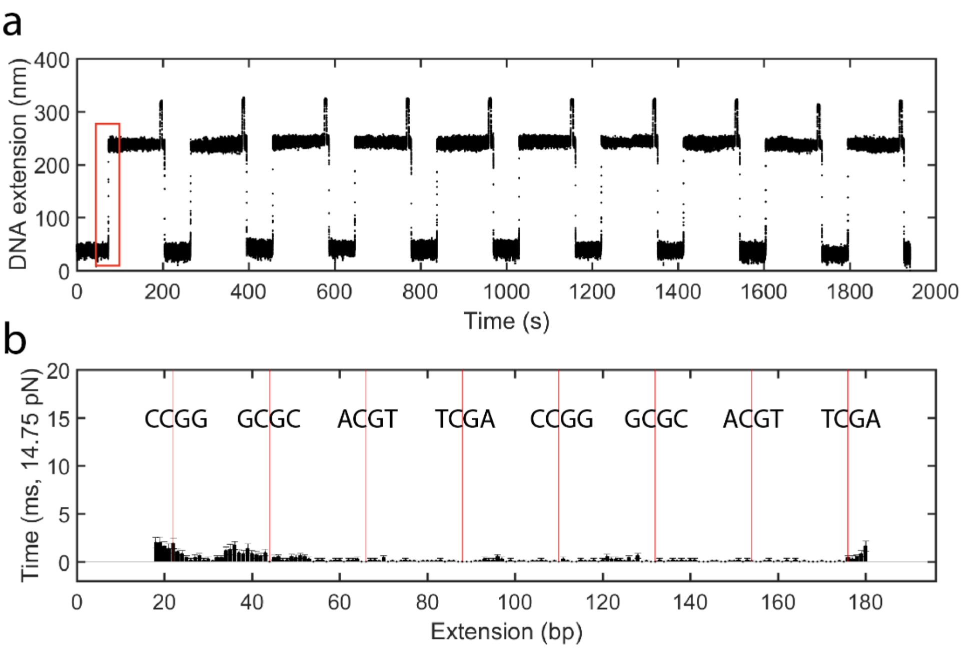

Figure S6. ATCG hairpin shows no pauses upon unfolded by a testing force of 14.75 pN

(a). Repetitive force-jumping assays to examine the melting of the ATCG hairpin at the absence of TET1 CXXC. The red window indicates the unfolding process of the ATCG hairpin. DNA extension is in the unit of a nanometer. Experimental conditions are the same as those in Figure 3.

(b). Overall pausing time distribution on the ATCG hairpin upon melting at the testing force. Data are from the regions indicated by the red window in (a). Red vertical lines indicate the positions of CpG sites. DNA extension is in the unit of bp. Error bars represent standard error. N = 47 traces from 6 molecules. Bin size = 1 bp.

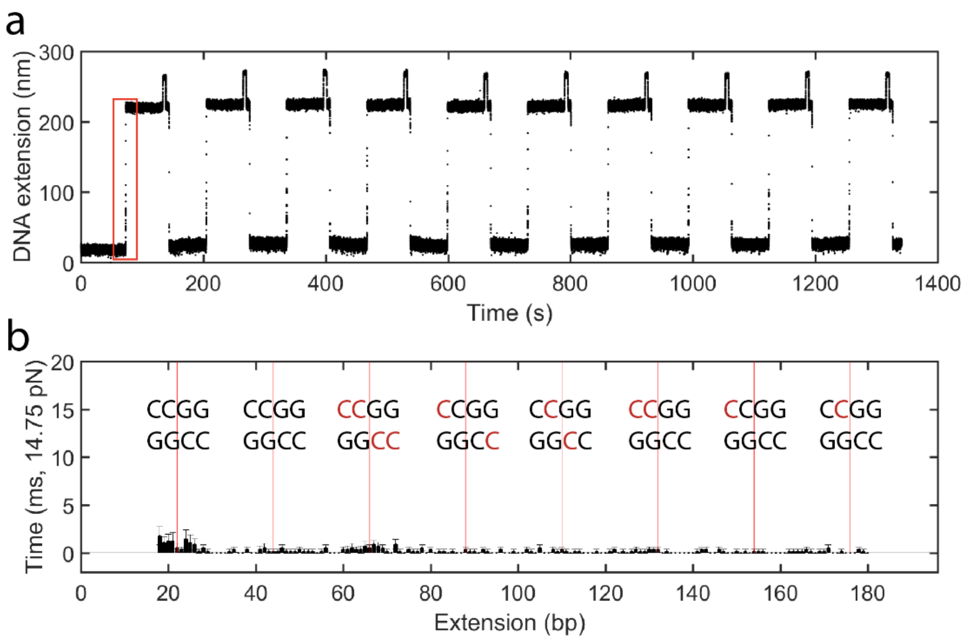

Figure S7. Hairpin of mCCGG shows no pauses upon unfolded by a testing force of 14.75 pN

(a). Repetitive force-jumping assays to examine the melting of the mCCGG hairpin at the absence of TET1 CXXC. The red window indicates the unfolding process of the hairpin. DNA extension is in the unit of a nanometer. Experimental conditions are the same as those in Figure 4.

(b). Overall pausing time distribution on the mCCGG hairpin upon melting at the testing force. Data are from the regions indicated by the red window in (a). Red vertical lines indicate the positions of CpG sites. Red Cs indicate the methylated cytosines. DNA extension is in the unit of bp. Error bars represent standard error. N = 28 traces from 4 molecules. Bin size = 1 bp.

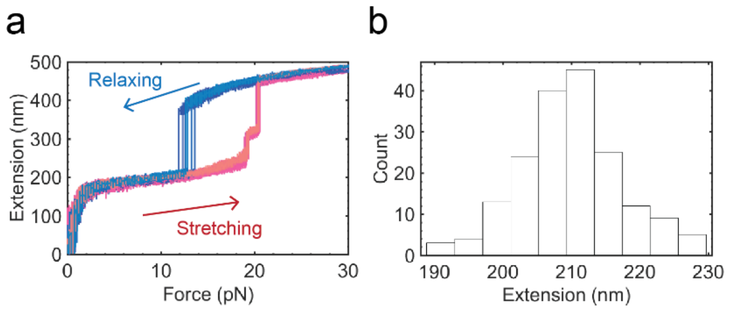

Figure S8. Unfolding and refolding of a CGI hairpin using single-molecule force spectroscopy.

(a). Mechanical manipulation of the CGI hairpin on single-molecule magnetic tweezers. The force loading rate is 3 pN/s.

(b). Histogram of changes in extension. The averaged change in extension is 210 ± 9 nm (mean ± sd, n = 180) regarding the entirely unfolded state.

(c). Histogram of unfolding forces. The averaged force is 20 ± 2 pN (mean ± sd, n = 180) regarding the entirely unfolded state.

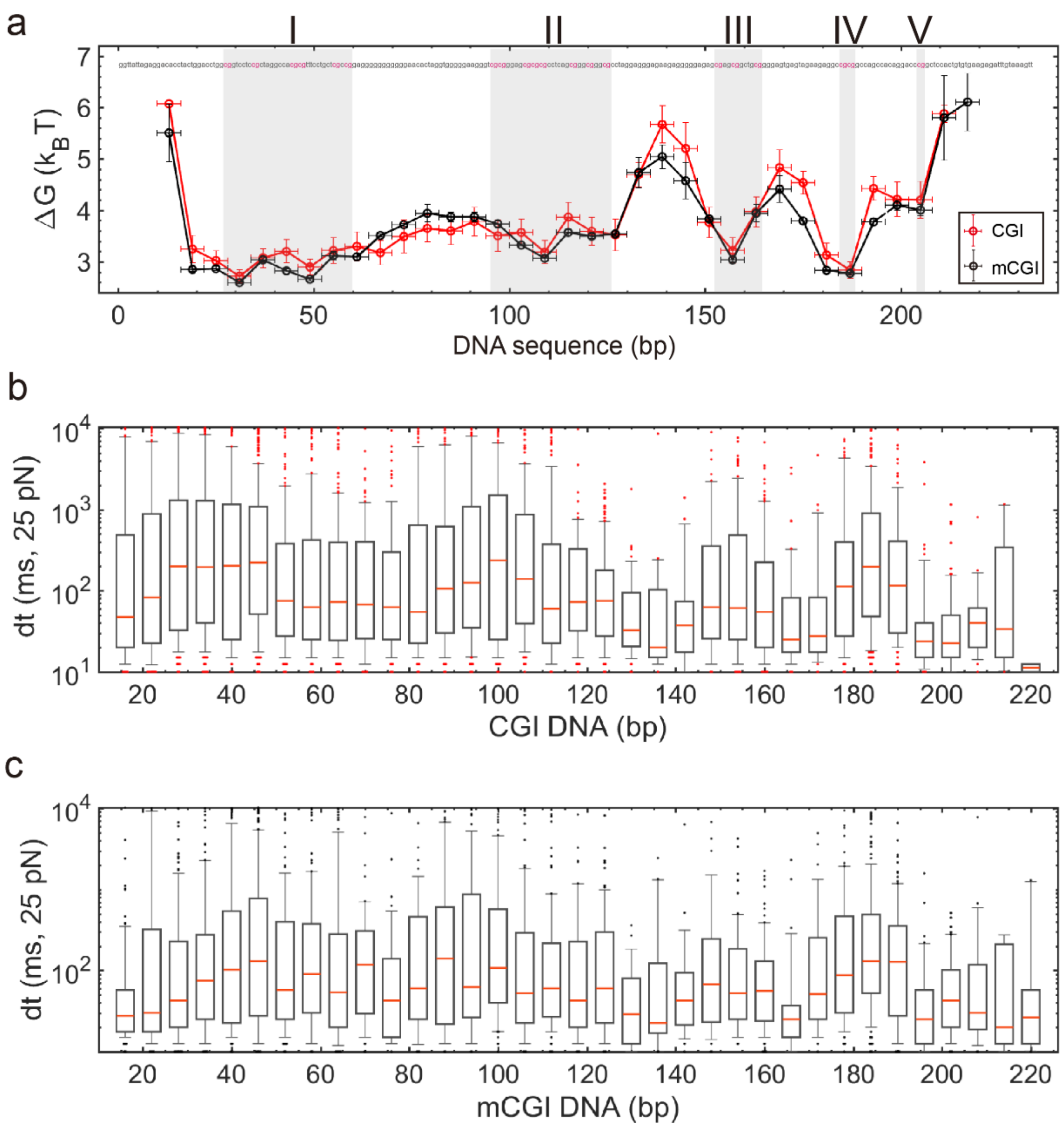

Figure S9. Energy landscapes and dissociation time of TET1 CXXC on the CGI/mCGI hairpins.

(a). Energy landscapes for TET1 CXXC binding the CGI hairpin (Red, n = 3899 traces) and the mCGI hairpin (Black, n = 2445 traces) (Mean ± sd).

(b). Dissociation time of TET1 CXXC from the CGI hairpin (N = 3899 traces, F_{test} = 25 pN). Red bar: Median. Box edges: the 25th and 75th percentiles. Whiskers: the 9th and 91th percentiles. Dots: Outliers of data.

(c). Dissociation time of TET1 CXXC from the mCGI hairpin (N = 2445 traces, F_{test} = 25 pN). Elements of box plot are the same as that in (b).
